# Genome-wide association studies dissect the genetic architecture of seed shape and size in common bean

**DOI:** 10.1093/g3journal/jkac048

**Published:** 2022-02-26

**Authors:** Willian Giordani, Henrique Castro Gama, Alisson Fernando Chiorato, Antonio Augusto Franco Garcia, Maria Lucia Carneiro Vieira

**Affiliations:** 1 Department of Genetics, ‘Luiz de Queiroz’ College of Agriculture, University of São Paulo, Piracicaba, SP 13418-900, Brazil; 2 Agronomic Institute of Campinas, Grains and Fibers Center, Campinas, SP 13075-630, Brazil

**Keywords:** *Phaseolus vulgaris*, grain morphology, GWAS, image-based phenotyping, association mapping

## Abstract

Seed weight and size are important yield components. Thus, selecting for large seeds has been a key objective in crop domestication and breeding. In common bean, seed shape is also important since it influences industrial processing and plays a vital role in determining the choices of consumers and farmers. In this study, we performed genome-wide association studies on a core collection of common bean accessions to dissect the genetic architecture and identify genomic regions associated with seed morphological traits related to weight, size, and shape. Phenotypic data were collected by high-throughput image-based approaches, and utilized to test associations with 10,362 single-nucleotide polymorphism markers using multilocus mixed models. We searched within genome-associated regions for candidate genes putatively involved in seed phenotypic variation. The collection exhibited high variability for the entire set of seed traits, and the Andean gene pool was found to produce larger, heavier seeds than the Mesoamerican gene pool. Strong pairwise correlations were verified for most seed traits. Genome-wide association studies identified marker–trait associations accounting for a considerable amount of phenotypic variation in length, width, projected area, perimeter, and circularity in 4 distinct genomic regions. Promising candidate genes were identified, e.g. those encoding an AT-hook motif nuclear-localized protein 8, type 2C protein phosphatases, and a protein *Mei2*-like 4 isoform, known to be associated with seed size and weight regulation. Moreover, the genes that were pinpointed are also good candidates for functional analysis to validate their influence on seed shape and size in common bean and other related crops.

## Introduction

Common bean (*Phaseolus vulgaris* L., 2*n *=* *2*x *=* *22) is the most widely consumed legume grain in the human diet (Broughton *et al.* 2003) and a valuable source of protein, energy, and micronutrients for more than 300 million people in the developing world (Gepts *et al.* 2008; Blair *et al.* 2010; Petry *et al.* 2015). Its relevance to global food security is becoming increasingly important due to markedly high population growth in the leading common bean consumption countries (Akibode and Maredia 2011; Bellucci *et al.* 2014) and the rising importance of protein resources of vegetable origin (Petrusán *et al.* 2016; Kumar *et al.* 2017). Brazil is one of the largest consumers and producers of common bean, harvesting around 3 million metric tons per year (FAOSTAT 2018).

Plant domestication can be understood as long-term experimentation involving the multifaceted relationships between environmental, anthropological, and evolutionary forces (Gepts 2004; Chacón-Sánchez 2018). In common bean, the occurrence of dual independent domestication events led to the establishment of 2 gene pools, the Mesoamerican and Andean. These gene pools display many morphological and genetic features (Singh *et al.* 1991b; Kwak and Gepts 2009), and especially traits related to a reduction in pod shattering and improvement in seed size, making *P. vulgaris* a good model for studies on crop evolution and domestication (Bitocchi *et al.* 2017). Accessions in the Mesoamerican gene pool exhibit greater genetic diversity (Mamidi *et al.* 2013) and are characterized by small-to-medium-sized seeds with “S” or “B” phaseolin types, while Andean seeds are larger, with “T,” “C,” “H,” and “A” phaseolin patterns (Gepts *et al.* 1986; Singh *et al.* 1991a).

Seed weight and size are important yield components and thus selecting for large seeds has been a key objective in crop domestication (Gavazzi and Sangiorgio 2017). A superficial consideration of the relationship between yield and seed size might suggest that these traits are positively correlated, since seed size should have a positive influence on yield. However, for common bean, in which hundred seed mass can range from 15 to over 60 g, strong negative correlations with yield have been reported (Adams 1967; Nienhuis and Singh 1986; White and González 1990), since heavier seeds result in fewer seeds per plant. This negative correlation has been of great interest to bean breeders because selecting for higher yields can produce accessions with small, lightweight seeds (White and González 1990). Nevertheless, on the major common bean markets such as Latin America, Africa, and Asia, larger seeds are more attractive to consumers and drive up market prices (Nienhuis and Singh 1986).

Seed shape is also an important commercial criterion for the processing industry, consumers, and farmers. For instance, information on bean morphological seed features such as length, width, thickness, projected area, perimeter, weight, circularity, and length-to-width ratio are critical for designing seed drills and systems for sorting, sizing, and handling (Mazhar *et al.* 2013; FAO and AfricaSeeds 2018; Shaw *et al.* 2020). Hence, bean breeders have become increasingly interested in understanding the genetic architecture of seed shape and size traits, mainly for identifying accessions that combine high yield, large seeds, adaptation to industrial processing, and consumer appeal (White and González 1990; Park *et al.* 2000; Blair *et al.* 2009; Lei *et al.* 2020).

Since the early days of genetics as a science, common bean seed traits such as weight and size were reportedly subject to quantitative inheritance (Johannsen 1911; Sax 1923), leading the botanist Wilhelm Johannsen to coin terms such as “genotype,” “phenotype,” and “gene”, and crucially providing evidence to resolve the classical biometric-Mendelian controversy (Roll-Hansen 1989). Further down the road, molecular markers were employed to dissect the genetic architecture of complex traits in plants based on 2 main approaches, known as quantitative trait locus (QTL) mapping and genome-wide association studies (GWAS) (Mitchell-Olds 2010; Xu *et al.* 2017). In family-based QTL mapping, designed crosses are used to unravel the genetic variations that differentiate progeny, in which meiotic cross-over events allow the identification of genetic loci segregating together with phenotypic variation (Lander and Botstein 1989). This methodology has been used to identify QTLs linked to many yield-related traits in common bean (González *et al.* 2017), including seed weight, size, and shape (Koinange *et al.* 1996; Park *et al.* 2000; Guzmán-Maldonado *et al.* 2003; Blair *et al.* 2009; Pérez-Vega *et al.* 2010; Checa and Blair 2012; Yuste-Lisbona *et al.* 2014; Geng *et al.* 2017).

The development of next-generation sequencing technologies significantly improved genotyping capacity and cut costs (Varshney *et al.* 2009), boosting the popularity of GWAS, which takes advantage of the historical accumulation of recombination events, obviating the need to synthesize segregating populations. This approach has enabled researchers to explore a wider genetic base and provide higher mapping resolution for studies focused on understanding the genetic underpinnings of quantitative traits (see Zhu *et al.* 2008; Korte and Farlow 2013; Huang and Han 2014; Giordani *et al.* 2021). Furthermore, high-throughput approaches involving image acquisition and analysis have also decisively favored GWAS, allowing large mapping populations, germplasm collections, and other breeding resources to be screened with progressively greater precision and accuracy for several phenotypic traits, including seed attributes (see Mir *et al.* 2019). Regarding bean seed size, Lei *et al.* (2020) applied GWAS to search for associations between 116 simple sequence repeat (SSR) markers and 4 seed traits. Although they were successful in finding marker–trait associations, the density of markers used was certainly not sufficient to represent all linkage disequilibrium (LD) blocks in the common bean genome. An essential step in this direction is to consider how LD decays across the common bean genome (Blair *et al.* 2018), especially when the biases ascribed to population structure and kinship are taken into account (Diniz *et al.* 2018). Therefore, there is still a scarcity of studies on combining GWAS with high-density single-nucleotide polymorphisms (SNPs) and high-throughput seed phenotyping to dissect common bean seed morphology, including not only seed size and weight but also other morphological aspects.

The aim of this study was to perform GWAS on a core collection of 180 common bean accessions with image-based phenotyping, and genotyping based on 10,362 SNPs to dissect the genetic architecture and identify genomic regions associated with 7 seed morphological traits related to weight (hundred seed mass), size (width, length, projected area, and perimeter), and shape (circularity and length-to-width ratio). Finally, we searched within genome-associated regions for candidate genes putatively involved in seed phenotypic variation.

## Materials and methods

### Plant material and growing conditions

This study was based on a core collection of 180 common bean accessions representing the genetic diversity of over 1,800 accessions from one of the largest Brazilian *P*. *vulgaris* germplasm collections at the Agronomic Institute of Campinas (IAC), Brazil (Perseguini *et al.* 2015). It is a well-known, high-variability repository for many traits of interest to breeders and consumers, such as seed morphology, biotic and abiotic stress resistance, and nutritional value, previously classified according to commercial group, origin, and type of phaseolin (see Perseguini *et al.* 2015; Diniz *et al.* 2018).

The seeds used for phenotypic evaluation were obtained from plants grown in the greenhouse in a fully randomized experimental design with 2 replicates, conducted in the State of São Paulo, Brazil (22°42′30″S, 47°38′00″W), during the 2017–2018 growing season (December–May). Each plot consisted of 1 pot containing 2 plants. Considering that all accessions were inbred lines grown under optimal conditions, high seed homogeneity was expected. Briefly, seeds were germinated in a biochemical oxygen demand (BOD) incubator at 26°C for 5 days prior to transplanting. Three seedlings were transferred to each 3 l pot containing soil and sand (3:1). After establishment, 1 plant was discarded from each pot. The remaining 2 were fertilized with 5 N–10 P–30 K according to crop recommendations and irrigated periodically until harvesting.

### Image-based acquisition and processing

In order to evaluate seed size, shape, and weight, 7 specific traits were measured on 50 seeds randomly selected from each plot. Seed size was determined by measuring seed length (LENGTH), width (WIDTH), perimeter (PERIM), and projected area (AREA). Length-to-width ratio (LWR) and circularity (CIRC) were calculated to reflect seed shape. Finally, seed weight was estimated on the basis of hundred seed mass (HSM).

**Figure 1.**
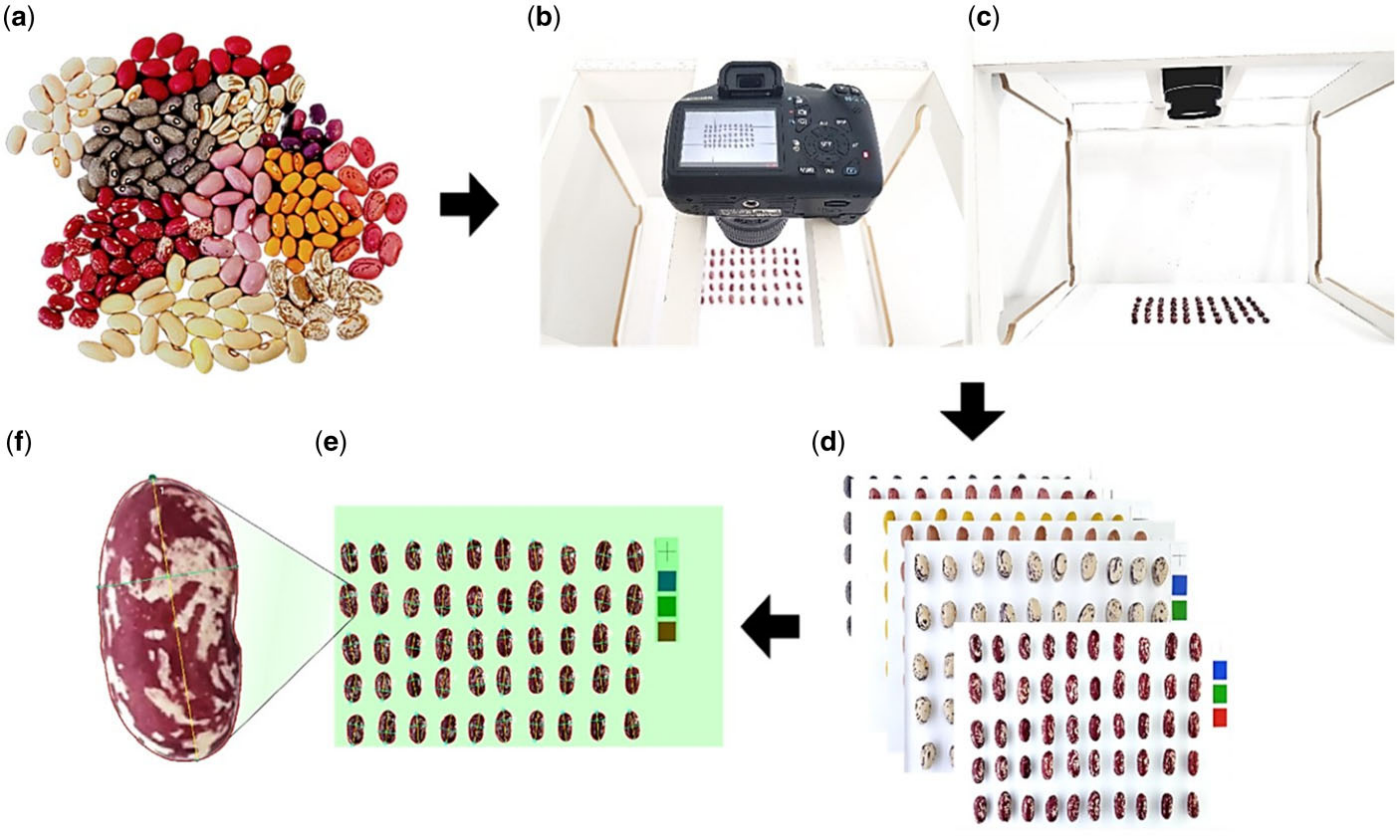
High-throughput image-based phenotyping for common bean seed size and shape. a) Representation of the phenotypic diversity of the IAC core collection panel. b) Top and (c) front view of the image acquisition station. d) Images of the seeds on a white background with a crossmark of 10 mm. e) Image after processing by SmartGrain software. f) Single seed measurement for shape parameters.

Seed size and shape properties were evaluated using high-throughput image processing. Seeds were photographed in a dark room with a supplementary light source and using a digital SLR camera (EOS 1100D, Canon, Tokyo, Japan) fixed perpendicularly on an image acquisition station, 35 cm above the seeds. The background color was either white or blue, to contrast with the tegument. A 10-mm crossmark was added to the background to convert from pixels to mm and indicate actual seed size on the image (Fig. 1). HSM in grams was determined on an analytical balance (Mark 1300, Technal, Brazil). A total of 18,000 seeds were phenotyped.

Images were analyzed using SmartGrain software (Tanabata *et al.* 2012), which can automatically detect every seed in the image and assess size and shape. It is recommended for high-throughput seed phenotyping in genetic mapping studies. The software processes the images rendering sequential coordinates along the seed perimeter. Briefly, the first step is to load the image and convert it from 24-bit (full color) to 1-bit (black and white) using a segmentation method. Next, the “cvErode” and “cvDilate” OpenCV functions are used to analyze seed morphology and exclude features such as awn, pedicel (for Poaceae), and funicle remnant (for Fabaceae). The outlines are then detected, all seeds labeled, and AREA, PERIM, and center of gravity computed using the “cvFindContour” function. The system automatically finds a set of perimeter coordinates for each seed in the image using: $P_{i}=\left(x_{i}y_{i}\right)\;\mathrm{for}\;i=0,\;1,\;\ldots ,\;n$; where the origin (0,0) for all $P_{i}$ is at the top-left corner of the image. The “cvContourArea” function then computes the area within the set of perimeter coordinates for each seed, and “cvArcLength” computes the PL (OpenCV Developers Team 2012). To calculate the LENGTH and WIDTH of each seed, the longitudinal and transversal axes are estimated from the set of perimeter points. For LENGTH, the algorithm detects the longest distance between 2 points on the perimeter coordinates and WIDTH is estimated based on the longest segment perpendicular to LENGTH. Finally, LWR and CIRC are computed using the following expressions:

$$\mathrm{LWR}=\frac{\mathrm{LENGTH}}{\mathrm{WIDTH}}$$

and

$$\mathrm{CIRC}=\;\frac{4\pi (\mathrm{AREA})}{{\mathrm{PERIM}}^{2}}.$$

In our study, all JPEG images were stored in a dedicated folder and batch analyzed. Finally, output images were manually checked to correct possible errors.

### Phenotypic data analysis

Linear mixed models were created to estimate the variance components and obtain adjusted means using restricted maximum likelihood (REML), implemented in ASReml‐R software (Butler *et al.* 2009):

$$Y_{ij}=\;\mu \;+\;g_{i(j)}\;+\;\rho_{j}\;+\varepsilon_{ij},$$

where $Y_{\mathrm{ijk}}$ is the phenotypic value of accessions i within gene pool j; μ is an intercept; $g_{i(j)}$ is the random effect of accessions i within gene pool j, where $g_{i(j)}∼N\left(0,\;\sigma_{g}^{2}\right)$; $\rho_{j}$ is the fixed effect of gene pool j; and $\varepsilon_{ij}$ is the experimental error associated with accessions i within gene pool j, where $\varepsilon_{ij}\;∼N\left(0,\;\sigma^{2}\right)$.

Likelihood ratio tests (LRT) were run to verify the statistical significance of the accession factor and the Wald test run to verify the significance of the gene pool. The best linear unbiased predictors were applied to compute the genotypic correlations using Pearson’s coefficient, and standardized to perform principal component analysis (PCA). Broad sense heritabilities for all 7 seed traits were estimated as the ratio of genotypic to phenotypic variance, expressed as a percentage using the formula: $H^{2}=\frac{{\hat{\sigma }}_{g}^{2}}{{\hat{\sigma }}_{g}^{2}+{\hat{\sigma }}_{e}^{2}}*\;100$, where ${\hat{\sigma }}_{g}^{2}$ is the genotypic variance and ${\hat{\sigma }}_{e}^{2}$ is the residual variance.

### Genotypic data

Genotyping by sequencing (GBS) had already been used to genotype the IAC core collection panel, as reported by Diniz *et al.* (2018). The procedures were carried out at the Genomic Diversity Facility of Cornell University’s Institute of Biotechnology. Briefly, DNA was extracted from young leaves using the DNeasy 96 Plant Kit (Quiagen, Hilden, Germany) and following the manufacturer’s protocol. Libraries were built using restriction enzyme ApeKI (5′ C/WGC 3′), and barcode adapters ligated to each individual sample. The samples were then pooled and amplified by PCR as described by Elshire *et al.* (2011), establishing 2 libraries in 95-plex. Single-end sequencing of up to 100 bp was run on the HiSeq 2500 platform (Illumina, San Diego, CA, USA) in a single lane. Sequences from the 2 libraries can be downloaded from the GenBank database, BioProject PRJNA336556, BioSamples SAMN05513252, and SAMN05513251. Bioinformatics procedures for SNP calling were performed by processing GBS raw sequences through the TASSEL-GBS pipeline, with the *P. vulgaris* (G19833) genome as reference (Schmutz *et al.* 2014). SNP data were filtered using the following selection criterion: minimum coefficient of inbreeding 0.9; minor allele frequency (MAF) ≥ 0.05; call rate < 0.9; and heterozygous loci set to missing data. A total of 10,362 SNPs were detected and are available in Diniz *et al.* (2018).

### GWAS and candidate gene search

GWAS on the 10,362 SNP markers and seed traits were performed using multilocus mixed modeling (MLMM; Segura *et al.* 2012), accounting for the kinship matrix, implemented in the GAPIT-R package (Lipka *et al.* 2012). In short, the model incorporates significant effects via a forward–backward stepwise approach. At each step, the variance components are re-estimated. If the newly included fixed effects are really influential, they might significantly reduce the residual variance and efficiently diminish the restrictions imposed by the mixed model on other markers correlated with population structure. This in turn improves statistical power and results in a lower false discovery rate compared to single-marker scanning and stepwise linear regression, especially if a conservative threshold such as Bonferroni (*α*/number of markers) is used. To avoid bias ascribed to double-shrinkage, best linear unbiased estimators (BLUEs) of the genotypic values for each trait were used in the association analysis.

The proportion of variance explained (PVE) by each association (SNP and seed trait) was calculated using the expression: *V*_QTL_ = *V*_pheno_, where *V*_QTL_ = 2freq (1 – freq)effect^2^ and *V*_pheno_ is the variance of the adjusted means for each trait.

The squared coefficient of correlation (*r*^2^) between the significant SNPs and neighboring SNPs (1 MB up and downstream) was computed in order to establish the LD block inherited together with the associated SNP, or inherited more frequently than could be expected by randomness. To do this, we used the LDheatmap R-package (Shin *et al.* 2006) and the expression below:

$$r^{2}=\frac{{{(P}_{\mathrm{AB}}-\;P_{A}P_{B})}^{2}}{P_{A}(1-P_{A})P_{B}(1-P_{B})},$$

where $P_{A}$ is the allelic frequency of A; $P_{B}$ is the allelic frequency of B, and $P_{\mathrm{AB}}$ is the allelic frequency of the AB haplotype. The genomic intervals investigated for candidate genes were then defined based on the LD block, as proposed by Assefa *et al.* (2019).

Protein sequences encoded by genes located in genomic intervals were obtained from the *P*. *vulgaris* genome (Schmutz *et al.* 2014), available in the NCBI Assembly database. For functional annotation, these sequences were subjected to a BLASTp search against the RefSeq database (O’Leary *et al.* 2016) by running Blast2GO v.5 (Conesa *et al.* 2005) with an *e*-value cutoff of 1 × 10^−5^ and a maximum of 20 hits. In addition, we used Gene Ontology (GO) mapping to assign GO terms by running Blas2GO with default parameters. Finally, aiming to assess the expression patterns of these genes during seed development, we consulted the common bean expression atlas (O’Rourke *et al.* 2014). The normalized expression data (reads/kb/million; RPKM) were obtained from 8 tissues: young flowers (FY), young pods (PY), pods approximately 9 cm long (PH), pods between 10 and 11 cm long (P1), pods between 12 and 13 cm long (P2), heart stage seeds (SH), stage 1 seeds (S1), and stage 2 seeds (S2). Moreover, in order to investigate relative gene expression among these tissues, we calculated the *Z*-score using: *Z* =(x-µ)/σ, where x is the RPKM value for each tissue; *µ* is the mean of RPKM values across tissues and σ the standard deviation. The application of this classical normalization allowed us to standardize RPKM values across a range of experiments, conditions and tissues, and contrast independently collected data (Cheadle *et al.* 2003).

## Results

### Phenotypic analysis

**Figure 2.**
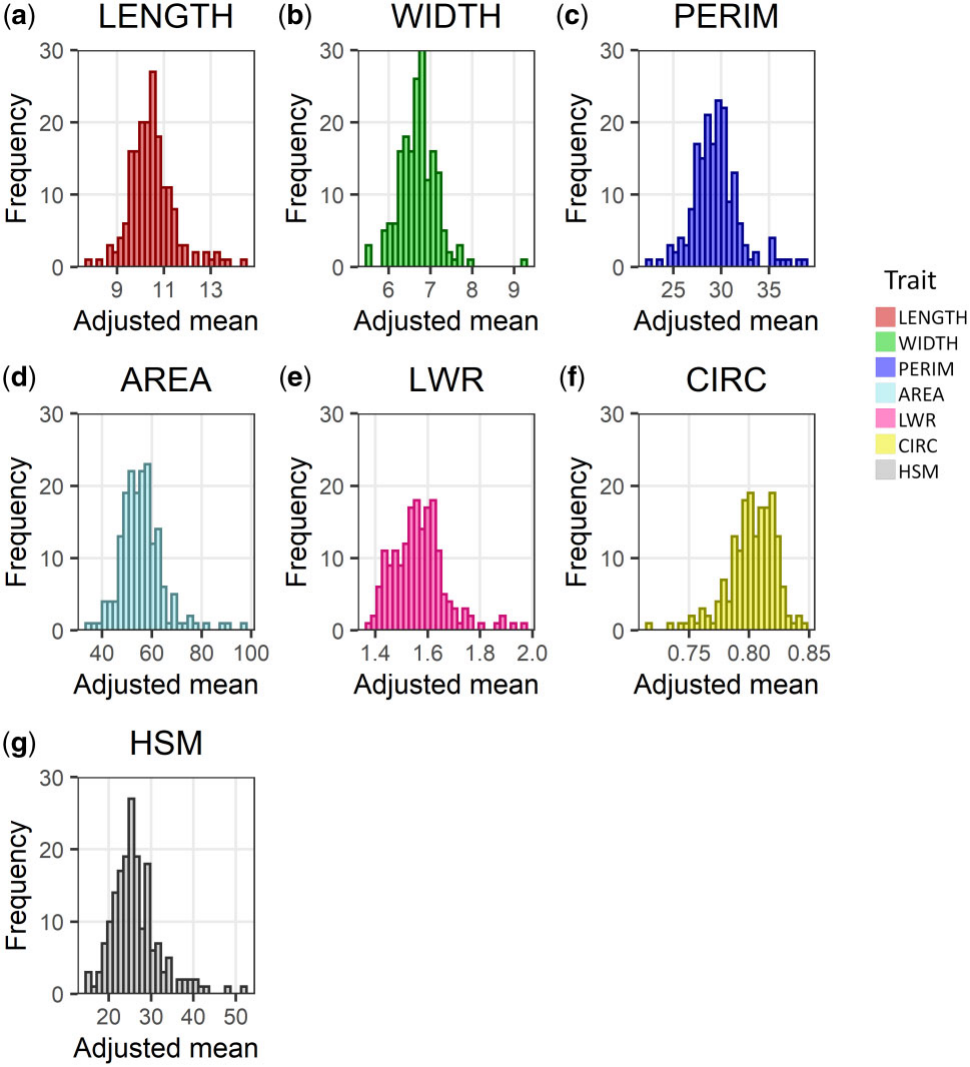
Frequency distribution of 7 seed traits, namely seed length (a; LENGTH); width (b; WIDTH); perimeter (c; PERIM); projected area (d; AREA); length-to-width ratio (e; LWR); circularity (f; CIRC); and hundred seed mass (g; HSM) for the 180 accessions of the IAC core collection panel.

Phenotypic evaluation of the core collection panel revealed wide variations across the whole set of seed traits (Fig. 2 and Supplementary Fig. 1). Low standard deviations for all seed size and shape traits were observed within each plot, mirroring the high seed homogeneity, and the quality of image-based phenotyping (Supplementary File 1). The descriptive statistics for LENGTH, WIDTH, PERIM, AREA, LWR, CIRC, and HSM are summarized in Table 1. Examining the dispersion of phenotypic data, broad amplitudes were found, especially for AREA, PERIM, and HSM. The coefficient of variation (CV) values between different replicates for all the traits were low to medium, ranging from 3.1% (CIRC) to 22.6% (AREA), indicating good experimental precision. The only traits with CVs > 10% were AREA and HSM, with respective values of 22.6% and 17.2%. Similarly, high broad-sense heritabilities (H2) were verified for all traits, varying from 75.9% (CIRC) to 90.9% (HSM). These results show that much of the observed phenotypic variation can be attributed to the genetic component, supporting the use of the core collection for GWAS purposes.

**Table 1.** Descriptive statistics, heritability, CV, and broad-sense heritability of 7 seed traits in the IAC common bean core collection.

Seed trait*a*	Amplitude	Overall mean	Mesoamerican mean	Andean mean	CV (%)	H² *b* (%)
LENGTH	7.5–15.0	10.5	10.3	11.2	10.0	87.2
WIDTH	5.1–9.5	6.7	6.68	6.8	7.7	82.8
PERIM	21.5–40.0	29.4	29.2	31.2	9.2	87.3
AREA	31.4–100.4	55.7	54.8	61.7	22.6	87.2
LWR	1.3–2.0	1.6	1.5	1.6	7.2	84.3
CIRC	0.65–0.85	0.80	0.80	0.79	3.1	75.9
HSM	16.6–55	26.2	25.4	30.9	17.2	90.9

a Seed length (LENGTH, in mm); width (WIDTH, in mm); perimeter (PERIM, in mm); projected area (AREA, in mm2); length-to-width ratio (LWR); circularity (CIRC); hundred seed mass (HSM, in g).

b Broad-sense heritability.

The suitability of the core collection for the GWAS was also supported by LRT, which confirmed a significant accession effect for the 7 seed traits, further evidence that genetic factors underlie common bean seed traits (Table 2). Of the 180 accessions evaluated, “Apetito Blanco,” “Branco Argentino,” and “Jalo 110” produced larger, heavier seeds, while some of the lowest values were found for “Sanilac,” “Rio Tibagi,” and “IAC Carioca Arua.” In terms of shape (CIRC and LWR), “Garbancillo,” “Sanilac,” and “Vermelhinho” exhibited the most circular seeds and “Red Kidney,” “Jalo,” and “Cal 143,” the most elongated (Supplementary Fig. 1).

**Table 2.** LRT and Wald test on random and fixed effects for 7 seed traits in the IAC common bean core collection panel.

Seed trait*a*	Gene pool effect		Accession effect			
	Wald test	*P*-value	LRT	*P*-value	Genetic	Residual
					variance	variance
					(%)	(%)
LENGTH	20.3	<0.0001	248.7	<0.0001	87%	13%
WIDTH	2.4	0.12	200.7	<0.0001	83%	17%
PERIM	17.6	<0.0001	250.8	<0.0001	87%	13%
AREA	15.1	<0.0001	246.9	<0.0001	87%	13%
LWR	16.8	<0.0001	215.2	<0.0001	84%	16%
CIRC	7.3	0.00698	145.3	<0.0001	83%	17%
HSM	23.1	<0.0001	246.3	<0.0001	91%	9%

a Seed length (LENGTH); width (WIDTH); perimeter (PERIM); projected area (AREA); length-to-width ratio (LWR); circularity (CIRC) and hundred seed mass (HSM).

**Figure 3.**
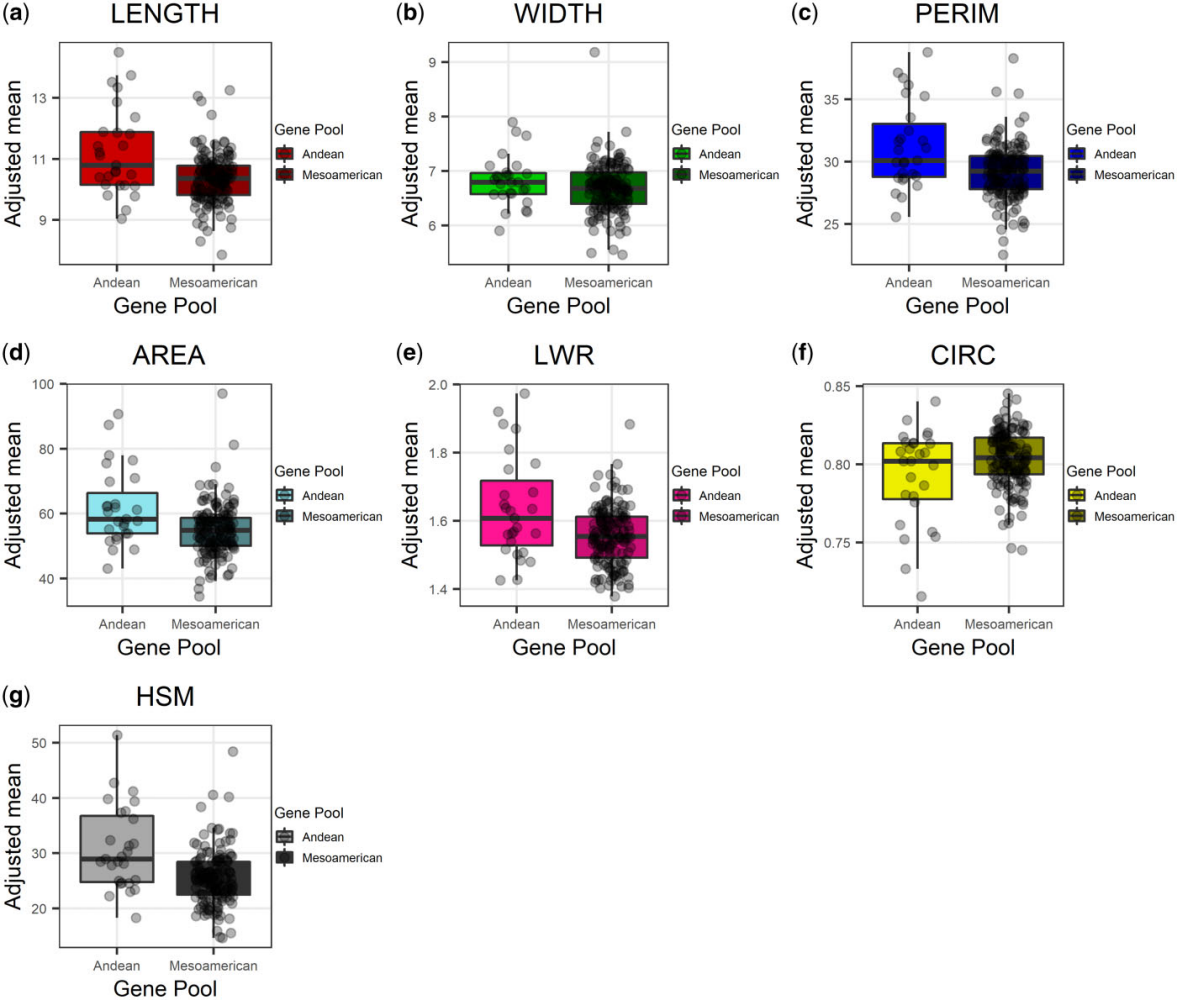
Boxplot of 7 seed traits, namely seed length (a; LENGTH); width (b; WIDTH); perimeter (c; PERIM); projected area (d; AREA); length-to-width ratio (e; LWR); circularity (f; CIRC); and hundred seed mass (g; HSM) for 180 accessions in the Andean and Mesoamerican gene pools.

Significant gene pool effects were also detected for all traits (*P* < 0.01) except WIDTH (*P =*** **0.12; Table 2), which could be due to slightly higher residual variance. However, for all sets of traits, low residual variances and high genetic variances were obtained, indicating wide genetic variability and high-accuracy phenotyping. The means for the Andean gene pool were higher than those for the Mesoamerican pool in respect of LENGTH, PERIM, AREA, LWR, and HSM, indicating larger, heavier seeds. On the other hand, the seeds produced by Mesoamerican accessions were more circular and less elongated (Table 1 and Fig. 3). It is important to note that, despite significant differences between gene pools, there was also considerable variation within the pools themselves (Figure 3 and Supplementary Fig. 1).

### Genotypic correlation and principal component analysis

**Figure 4.**
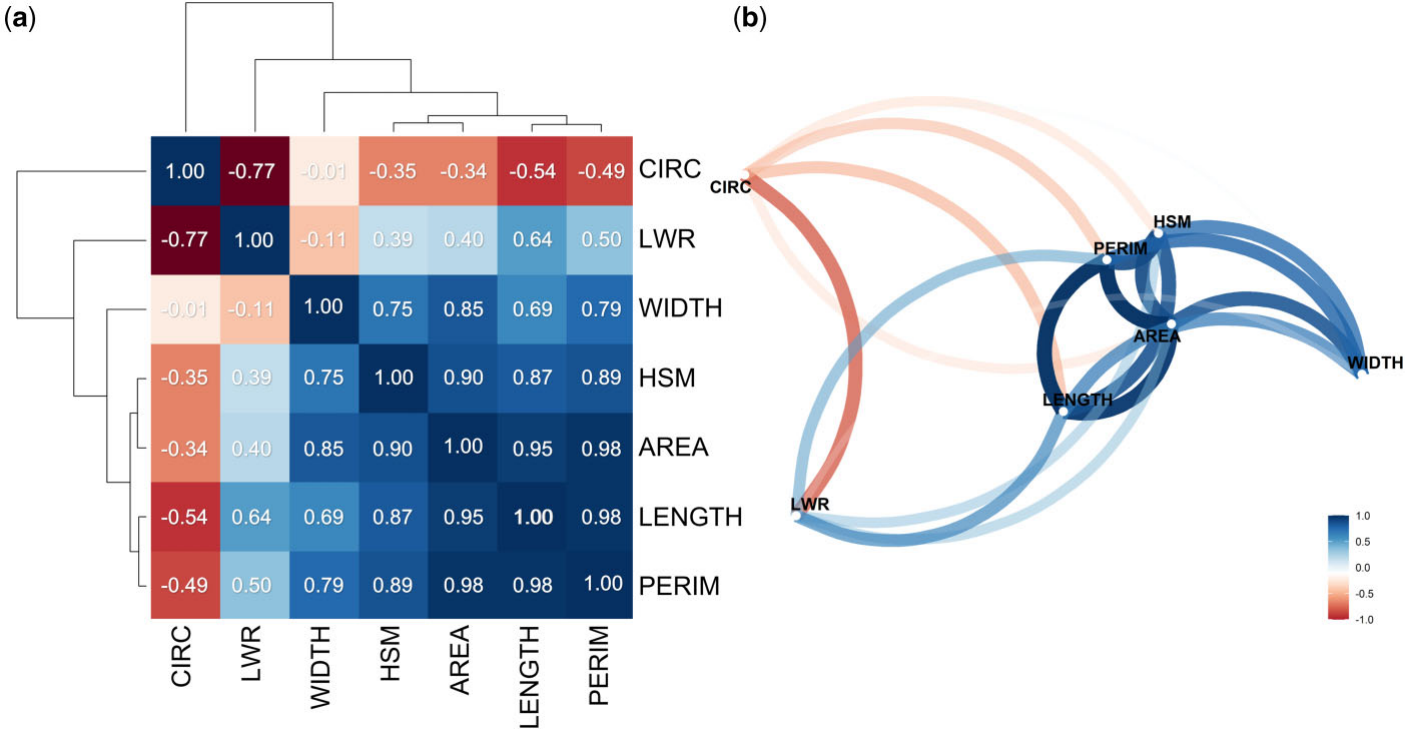
Heatmap of pairwise Pearson correlation coefficients (a) and network plot (b) of 7 seed traits: seed length (LENGTH); width (WIDTH); perimeter (PERIM); projected area (AREA); length-to-width ratio (LWR); circularity (CIRC); and hundred seed mass (HSM) in the 180 accessions of the IAC core collection panel.

Pairwise genetic correlation analysis enabled us to detect at least 3 groups of correlated traits. Pearson coefficients were strongly positive between all pairs of seed size traits, including weight (Fig. 4, a and b). Among these traits, correlations varied from 0.69 (WIDTH–LENGTH) to 0.98 (AREA–PERIM). For instance, the strongest correlations were found between PERIM–AREA, PERIM–LENGTH, and AREA–LENGTH (0.98, 0.98, and 0.95, respectively). In terms of seed shape (LWR and CIRC), distinct behavior was detected; even though they showed strong negative correlation with each other (−0.77), correlations with the remaining traits differed, as shown by the network plot (Fig. 4b). LWR exhibited moderately positive correlations with LENGTH, PERIM, AREA, and HSM, and a weak negative correlation with WIDTH. In contrast, CIRC exhibited moderately negative correlations with all seed traits except WIDTH, which was close to zero (Fig. 4).

**Figure 5.**
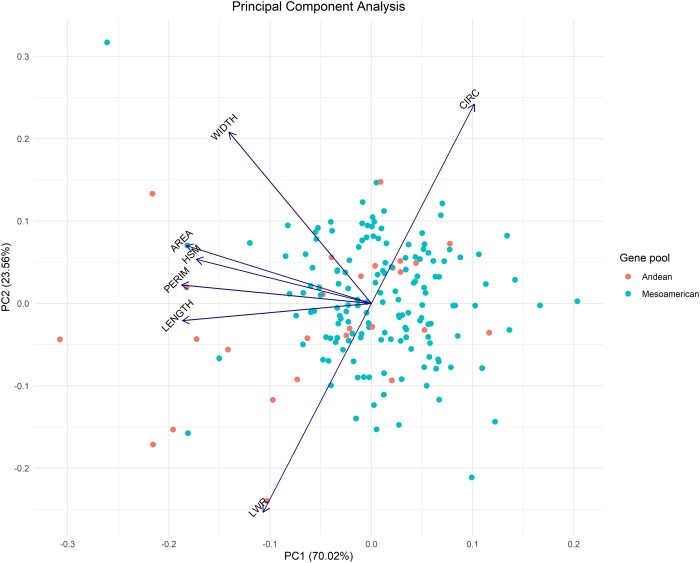
PCA of the IAC common bean core collection panel based on the phenotypic variability of 7 seed traits: seed length (LENGTH); width (WIDTH); perimeter (PERIM); projected area (AREA); length-to-width ratio (LWR); circularity (CIRC); and hundred seed mass (HSM). Orange and blue dots indicate accessions of Andean and Mesoamerican origin, respectively.

PCA was performed to ascertain the key sources of variation in the set of seed traits and to check whether the 7 evaluated traits were consistent for differentiating the accessions according to gene pool origin. The first 2 principal components (PC) accounted for 93.58% of the variation, in which PC1 mainly described the variation in LENGTH, PERIMETER, AREA, WIDTH and HSM, and PC2 captured most of the variance in CIRC and LWR (Fig. 5), corroborating the correlation clusters verified by the Pearson coefficient (Fig. 4b). The strong negative correlation between CIRC and LWR can also be detected by PCA, since the loadings representing these traits point in opposite directions. Finally, although it was not possible to clearly cluster the gene pools, most of the accessions that stood out in terms of AREA, HSM, PERIM, LENGTH, and LWR were of Andean origin.

### Genome-wide association studies

**Figure 6.**
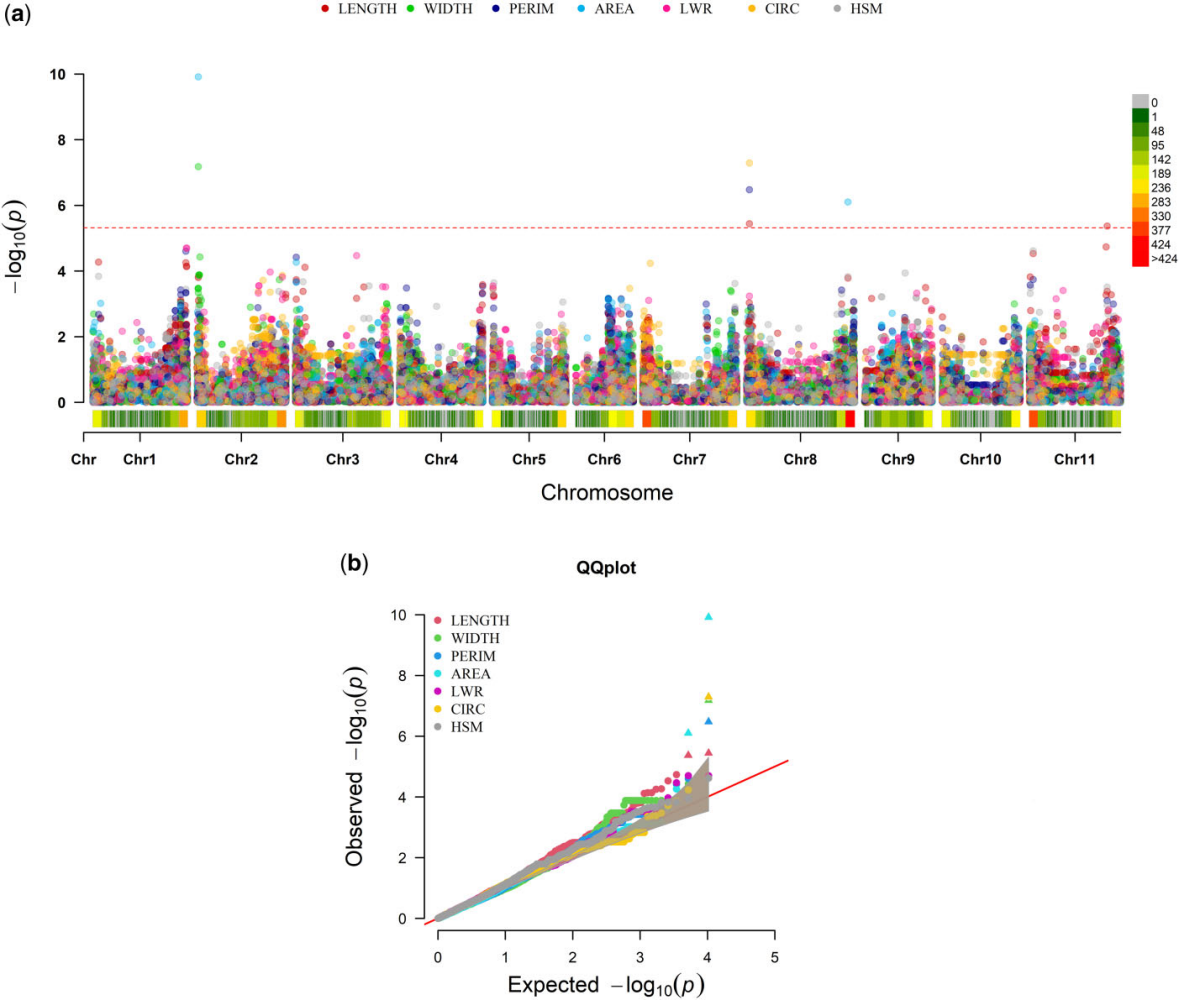
GWAS on 7 common bean seed traits for the 180 accessions of the IAC core collection panel. Seed length (LENGTH) is shown in red; width (WIDTH) in green; perimeter (PERIM) in blue; projected area (AREA) in light blue; length-to-width ratio (LWR) in pink; circularity (CIRC) in yellow and hundred seed mass (HSM) in gray. The heatmap bars in the Joint Manhattan plot (a) depict SNP density along the 11 *P. vulgaris* chromosomes, and the dashed line indicates the Bonferroni threshold (*P* < 4.82E-06). In the QQ plot (b), the shaded area indicates a 95% confidence interval.

GWAS for the 7 seed traits were conducted on a total of 10,362 SNPs. As shown in the quantile–quantile (QQ) plots (Fig. 6b), according to the MLMM model the observed *P*-values are close to the expected values under the null hypothesis for all traits, indicating that there were no systematic false positive associations.

Seven marker–trait associations with *P*-values below the Bonferroni threshold (*P* < 4.82E-06) were considered significant for LENGTH, WIDTH, AREA, PERIM, and CIRC, and were located in 4 distinct genomic regions of 3 chromosomes (Pv02, Pv08, and Pv11; Fig. 6). Of the 7 associations, 2 SNPs were associated with more than 1 trait. For LENGTH, the 2 significantly associated SNPs (S1_325844152 and S1_507205534) were positioned on loci 1797591 and 42751382 bp of chromosomes Pv08 and Pv11, respectively. These SNPs had effects of similar magnitude (0.69 and −0.67 mm) and explained 5.94% and 6.39% of the phenotypic variation. SNP S1_325844152 was also found to be associated with PERIM and CIRCULARITY. For PERIM, it accounted for 7.16% of the phenotypic variation, while for CIRC the phenotypic variation explained (PVE) by the association was as high as 17.6% (Table 3, Fig. 6, and Supplementary Fig. 2).

**Table 3.** Single-nucleotide polymorphisms (SNP) significantly associated with seed length, width, area, perimeter, and circularity, according to the GWAS results for the IAC core collection panel.

Seed trait^*a*^	Marker name	Chr^*b*^	Position (bp)	*P*-value	MAF	Allelic reference	Allelic variant	Variant allele effect	PVE^*c*^ (%)
LENGTH	S1_325844152	Pv08	1797591	3.61E-06	0.07	T	C	0.69	5.94
	S1_507205534	Pv11	42751382	4.28E-06	0.08	G	A	−0.67	6.39
WIDTH	S1_53011276	Pv02	805645	6.64E-08	0.11	A	C	0.55	23.98
AREA	S1_53011276	Pv02	805645	1.22E-10	0.11	A	C	10.86	23.3
	S1_379992973	Pv08	55946412	7.91E-07	0.08	C	T	6.01	5.18
PERIM	S1_325844152	Pv08	1797591	3.36E-07	0.07	T	C	1.95	7.16
CIRC	S1_325844152	Pv08	1797591	5.11E-08	0.07	T	C	−0.023	17.6

SNPs significantly associated according to the Bonferroni multiple test correction (*P* < 4.82E-06).

a Seed length (LENGTH); width (WIDTH); perimeter (PERIM); projected area (AREA); and circularity (CIRC).

b Chr, chromosome.

c PVE, phenotypic variation explained by the SNP–trait association. For the Variant allele effect, negative values indicate that the allelic variant is associated with lower phenotypes.

AREA-associated SNPs (S1_53011276 and S1_379992973) were located on Pv02 and Pv08 at 805645 bp and 55946412 bp, respectively. For this trait, the effect of S1_379992973 was 6.01 mm^2^ with a PVE of 5.18%, while S1_53011276 explained an even higher portion of the phenotypic variation (23.3%), with an additive effect of 10.86 mm^2^, resulting mainly from its influence on seed width. For WIDTH, this SNP also accounted for 23.7% of the total phenotypic variation and the effect of the variant allele on this trait was 0.55 mm. No significant associations were detected for LWR and WEIGHT (Table 3 and Fig. 6).

For the 4 significantly associated SNPs, both the variant and the reference allele were found in both gene pools. However, the minor allele variant always occurred at more balanced frequencies in both gene pools. Corroborating the phenotypic analysis which revealed larger seed size in the Andean accessions than in the Mesoamerican accessions, the alleles with positive effects were also present at higher frequencies in the Andean gene pool, except for S1_507205534 (Table 3 and Supplementary Fig. 3).

### Candidate genes

With the aim of delimitating the genomic window for candidate gene investigation, LD analysis was performed on the borders of the significant SNP–seed trait associations. The genomic intervals varied between 358.4 kb for S1_53011276 and 558.6 kb for S1_507205534 (Supplementary Fig. 4). Supplementary Table 1 gives a complete list of 192 genes predicted in the set of associated regions, 188 of which were annotated; 50 in the genomic region harboring SNP S1_53011276; 64 in S1_325844152; 45 in S1_325844152; and 29 in S1_507205534. To give a clearer picture of the potential functions of these genes, functional groups were classified based on the GO database. Among the inferred genes, 32 had no functional annotation or were classified as protein-coding genes of unknown function.

In order to narrow down the number of candidate genes and obtain further evidence of their importance in regulating common bean seed size and shape, we consulted the *P. vulgaris* expression atlas for 8 tissues and stages related to seed development (O’Rourke *et al.* 2014), extending from flowering to late phase seed formation. Among the 188 annotated genes, 156 were expressed in one or more tissues or stages and 13 were considered the most promising (Table 4 and Supplementary Table 2) with homologs already reported as putatively involved in seed development or related traits in crops or model species.

**Table 4.** Candidate genes identified in GWAS-associated genomic regions able to modulate seed shape and size in common bean.

Seed trait^*a*^	Associated SNP	Gene ID	Functional annotation	Genomic location
WIDTH and Area	S1_53011276	Phvul.002G004400	Pentatricopeptide repeat-containing protein	Pv02:765463…766998
		Phvul.002G006100	Phosphatase 2C 29-like protein	Pv02:516568…518904
		Phvul.002G006700	AT-hook motif nuclear-localized protein 8	Pv02:656431…661984
		Phvul.002G007100	Transcriptional regulator SUPERMAN	Pv02:698486…703324
LENGTH, PERIM, and CIRC	S1_325844152	Phvul.008G019500	*Mei2*-like 4 isoform X1 protein	Pv08:1727505…1734392
		Phvul.008G020600	Crowded nuclei 1 protein	Pv08:1626867…1635158
		Phvul.008G022600	Pentatricopeptide repeat-containing protein	Pv08:1854380…18556627
		Phvul.008G023900	Pentatricopeptide repeat-containing protein	Pv08:1974646…1978175
		Phvul.008G024300	Pentatricopeptide repeat-containing protein	Pv08:1999723…2003178
		Phvul.008G024400	Pentatricopeptide repeat-containing protein	Pv08:2004322…2008280
AREA	S1_379992973	Phvul.008G244300	Putative B3 domain-containing protein	Pv08:59274246…59279462
LENGTH	S1_507205534	Phvul.011G160400	Cup-shaped cotyledon 2-like protein	Pv11:45614432…45616861
		Phvul.011G163100	Ribosomal protein L11 methyltransferase	Pv11:46181028…46188144

Significantly associated SNPs according to the Bonferroni multiple test correction (*P* < 4.82E-06).

a Seed length (LENGTH); width (WIDTH); perimeter (PERIM); projected area (AREA); and circularity (CIRC).

In the LD block containing SNP S1_53011276, associated with WIDTH and AREA, several genes could be pinpointed as promising candidates (Table 4). S1_53011276 is positioned within an exon of Phvul.002G007100, which encodes the *SUPERMAN* transcriptional regulator, well known for playing a role in *Arabidopsis* reproductive organ morphology (Sakai *et al.* 1995, 2000). In terms of the *Z*-score, substantial negative values were observed for this gene in most tissues, indicating that compared to the whole set of genes and tissues analyzed herein, Phvul.002G007100 was the one with lowest general expression (Supplementary Fig. 5). Note that the expression of this gene was verified at the 3 stages of seed development (SH, S1, and S2), with stronger expression in the 2 later stages (S1; RPKM = 69 and S2; RPKM = 53). Likewise, in this genomic region, Phvul.002G004400 encodes for a pentatricopeptide repeat-containing protein, already reported as a prominent player in seed development in various crops (Gutiérrez-Marcos *et al.* 2007; Liu *et al.* 2013a; Li *et al.* 2014; Verma *et al.* 2015) and highly expressed at the initial stage of seed development (SH; RPKM = 122) (Supplementary Table 2). Phvul.002G006100 is annotated as a protein phosphatase 2C 29-like gene and exhibited remarkably strong expression in all the 8 evaluated tissues, with RPKM varying from 111 in P1 to 2029 in SH, and particularly high *Z*-scores for SH (1.22), S1 (1.57), and S2 (1.66) (Supplementary Table 2 and Supplementary Fig. 5). Finally, Phvul.002G006700 is a gene predicted as an AT*-*hook motif nuclear-localized protein 8 which has been shown to underlie seed size in other species (Sharma Koirala and Neff 2020), with high RPKM values for FY, PH, and the 3 seed stage tissues (Supplementary Table 2).

Associated with LENGTH, PERIM, and CIRC, SNP S1_325844152 is located within an exon of Phvul.008G020600, a gene that encodes a crowded nuclei 1 protein*.* Consistent with the expression atlas, this gene exhibited the greatest expression for FY, PV, PH, P1, P2, and SH, and also the highest *Z*-scores for most tissues, especially in the early developmental stages (Supplementary Fig. 5). In the same haplotype block, a homolog of Phvul.008G019500, a protein *Mei2*-like 4 isoform, was recently reported as a modulator of grain size and weight in *Oryza sativa* (Lyu *et al.* 2020) and was highly expressed in common bean during the early seed developmental stage (SH; RPKM = 488). In addition, this region comprises several genes encoding for pentatricopeptide repeat-containing proteins (Phvul.008G022600, Phvul.008G023900, Phvul.008G024300, and Phvul.008G024400), and Phvul.008G022600 and Phvul.008G024400 are of particular note for their high RPKM values along all tissues examined herein (Supplementary Table 2). Furthermore, in Pv08, significant for AREA, the S1_379992973 LD region contains a putative B3 domain-containing protein (Phvul.008G244300) that is worthy of attention since the B3 domain has been found in transcription factors involved in seed development control (Swaminathan *et al.* 2008). Finally, SNP S1_507205534, significantly associated with LENGTH, is positioned in an exonic region of Phvul.011G163100, a ribosomal protein L11 methyltransferase whose expression is highlighted in SH (RPKM = 59) and S1 (RPKM = 48) tissues (Supplementary Table 2). This SNP has an LD with a genomic locus that harbors other promising candidate genes, such as Phvul.011G160400, a cup-shaped cotyledon 2-like protein with high expression in FY (RPKM = 205) and PH (RPKM= 275) tissues (Supplementary Table 2).

## Discussion

### Variance components and heritabilities

In common with the majority of crops, breeding programs for common bean are primarily aimed at releasing high-yield cultivars with resistance to biotic and abiotic stresses (Miklas *et al.* 2006; Beaver and Osorno 2009; Singh and Schwartz 2010; Assefa *et al.* 2019). However, bean breeders face additional challenges since they need to pay close attention to the end consumers’ needs (Kelly and Miklas 1998; Santalla *et al.* 2004; Beaver and Osorno 2009; Assefa *et al.* 2019). Therefore, understanding the genetic architecture of key seed morphological traits is a critical step toward the development of cultivars combining high yield, industrial compatibility, and consumer acceptance (White and González 1990; Park *et al.* 2000; Blair *et al.* 2009; Lei *et al.* 2020). In addition, since seed size has played a crucial role in common bean domestication, identifying the genes responsible for phenotypic variation can help us understand the genetic basis of adaptation (Chacón-Sánchez 2018).

Herein we studied the genetic architecture of 7 common bean seed traits including those related to size, shape, and weight. We used a core collection panel derived from one of the largest Brazilian germplasm collections. Despite its modest size, the panel embodies broad genetic diversity (see Perseguini *et al.* 2015 and Diniz *et al.* 2018) and is considered a high-variability repository for many important traits, including seed morphology and composition, and resistance to biotic and abiotic stresses. Furthermore, this panel was recently used to successfully map genomic regions associated with the common bean’s response to root-knot nematodes (Giordani *et al.* 2021), further evidence of its suitability for GWAS.

We observed wide variability for all the traits analyzed and coefficients of variation were predominantly low (<10%), indicating that image analysis of seed properties leads to a slight experimental error, as reported by Fıratlıgil-Durmuş *et al.* (2010). Significant accession effects were observed for all traits, and combined with high broad-sense heritabilities (>75.9%), show that a substantial portion of phenotypic variation might be ascribed to the genetic component, highlighting its importance in the expression of these quantitative traits, and thus lending weight to the use of this core collection for GWAS (Table 1). These results corroborate the findings of other studies on bean seed morphology, also reporting high heritabilities (Motto *et al.* 1978; Yuste-Lisbona *et al.* 2014; Rana *et al.* 2015; Singh *et al.* 2018; Wu *et al.* 2020).

Even though both domestication events increased seed size and weight compared to their wild ancestors (Gepts 2004; Chacón-Sánchez 2018), it is a fact that Andean accessions produce larger seeds than Mesoamericans (Gepts and Bliss 1988; Singh *et al.* 1991b; Logozzo *et al.* 2007; Lei *et al.* 2020). We once again confirmed the larger seed size of Andean accessions based on LEGNTH, PERIM, AREA, and HSM. No significant differences in WIDTH (*P* = 0.12) were exhibited by the individual gene pools. These findings suggest that the main reasons for the seed size discrepancy between the 2 pools are related to the shape aspect of longitudinal development, an observation supported by the higher LWR of the Andean seeds and the higher CIRC values for Mesoamerican accessions.

### Pairwise correlations and principal component analysis

Corroborating other evidence, the core collection herein evaluated showed strong positive pairwise correlations among seed size traits (Pérez-Vega *et al.* 2010; Rana *et al.* 2015; Herron *et al.* 2020; Lei *et al.* 2020; Murube *et al.* 2020). We verified that the overall size, considering both weight and projected area, was influenced principally by an increase in the length dimension. Even though less pronounced, we observed a negative correlation between CIRC and all the other size traits except WIDTH, suggesting that small seeds are usually rounder while larger seeds tend to be elongated. Hence, our results show that an extension in LENGTH does not necessarily imply an increase in the distance between one side and the other at the hilum region. Consequently, since WIDTH shows lower genetic variance (Table 2), LENGTH is suggested to be the main seed size parameter affecting shape proprieties. PCA provided further insights into the relationships among seed traits. PC1 accounted for over 70% of total variability, explaining mainly the variation in seed size traits, while for PC2, the most prevalent traits were related to seed shape. Although PCA did not clearly group the gene pools, the majority of the accessions positioned further away from the intersection of the biplot were found to be Andean.

#### SNP–trait associations

The GWAS approach is fundamentally based on LD and aimed at identifying genetic markers physically linked to the actual causal variant for a given trait. Nonetheless, several other factors over and above physical linkage may influence LD estimates. In breeding programs, intense selection, admixture of populations and crossing using a limited number of elite genotypes reduces genetic diversity and may strongly impact LD patterns and consequently affect GWAS resolution and statistical power (Delfini *et al.* 2021). In general, LD decay is slower in autogamous species, such as common bean, in which recombination is less effective than in allogamous species (Flint-Garcia *et al.* 2003).

In the populations typically used for GWAS, related individuals share both causal and noncausal alleles, and thus LD between these loci can result in spurious associations (Korte and Farlow 2013). For this reason, kinship and population structure are usually modeled in the association analysis (Zhao *et al.* 2007; Zhang *et al.* 2010). For the core collection used herein, our group has previously studied LD showing that kinship bias is heavier than the bias ascribed to population structure. We proved that when kinship is taken into account, population structure bias is no longer observed in the collection (Diniz *et al.* 2018). Additionally, we have shown that controlling these biases, LD in *P. vulgaris* decays to 0.1 at a distance of 1 Mb, indicating that LD for this collection is fairly widespread, in contrast to that observed in cultivars of soybean (133 kb) (Zhou *et al.* 2015) and rice (123 kb) (Huang *et al.* 2010).

Although it has been shown that LD is not uniformly distributed across the genome, the overall slower LD decay indicates that the SNP density used in this study could be adequate for tagging most of the LD blocks. However, we expected to find noncausal alleles among the significant associations. For this reason, our strategy of defining the entire loci in disequilibrium harboring the significantly associated SNPs was imperative for finding candidate genes.

In the present study, we have confirmed the previous results and demonstrated that, for all seed traits, when fitting kinship to the MLMM models, *P*-values exhibit low general inflation, since they closely adhered to the expected results under null hypothesis, suggesting goodness of fit and low false discovery rates (Fig. 6b).

In terms of the genetic architecture of seed size and shape, our results indicate that, although exhibiting quantitative behavior, some genomic regions do account for a considerable amount of variation. GWAS analysis allowed us to identify marker associations with moderate to high effects in 4 chromosome regions. On Pv02 at 805645 bp, S1_53011276 was significantly associated with WIDTH and AREA and explained over 23% of the phenotypic variance. This SNP is a promising candidate for marker-assisted selection, especially since the beginning of chromosome Pv02 has also been reported as linked to seed width (Geng *et al.* 2017, Wu *et al.* 2020; Geravandi *et al.* 2020) and other seed traits (Blair *et al.* 2006; Pérez-Vega *et al.* 2010; Lei *et al.* 2020; Murube *et al.* 2020). Our findings also endorse Pv08 as a key chromosome underlying the size and shape aspects of common bean seeds. At positions 1797591 and 55946412 bp, 2 SNPs (S1_325844152 and S1_379992973) were associated with 4 traits (LENGTH, AREA, PERIM, and CIRC). Among these associations, the high PVE (17.6%) found between S1_325844152 and CIRC is particularly significant. Remarkably, this chromosome has already been reported to harbor seed size QTLs (Park *et al.* 2000; Blair *et al.* 2006; Pérez-Vega *et al.* 2010; Wright and Kelly 2011; Geravandi *et al.* 2020). Since there are still no genetic mapping studies for CIRC and considering that it exhibits a marked influence on LENGTH, the significant effect of QTLs previously reported for seed length (Park *et al.* 2000; Pérez-Vega *et al.* 2010) not only confirm the role of Pv08 in determining LENGTH but also suggest that it does contribute to CIRC. Finally, at position 42751382 bp of Pv11, the association between LENGTH and SNP S1_507205534 corroborates previous findings reporting QTLs underlying seed length at a similar location on the same chromosome (Park *et al.* 2000; Murube *et al.* 2020).

In common bean, dual domestication significantly impacted traits related to a reduction in pod shattering and improvement in seed size. These traits merit singular attention because of their role in early adaptation and importance for plant breeding (Chacón-Sánchez 2018).

Regarding the differences between the 2 gene pools, it is noteworthy that the 4 associated SNPs are segregating in both pools (Supplementary Fig. 3). This suggests that such informative markers can be useful for improving genotypes of both origins using marker-assisted selection, and consequently afford good opportunities for breeding, since crossing Andean and Mesoamerican accessions is typically difficult.

#### Candidate genes

In order to define the genomic window on which to base the search for candidate genes, loci spanning significant associations were investigated based on LD (Supplementary Fig. 4). Of the 192 genes predicted in those regions, 188 were annotated and 156 classified against the GO database (Supplementary Table 1) and expressed in one or more tissues related to seed development according to the *P*. *vulgaris* expression atlas (O’Rourke *et al.* 2014). Herein, we have highlighted some of the most promising candidates that may influence WIDTH, AREA, LENGTH, PERIM, and CIRC.

For WIDTH and AREA, homologs of Phvul.002G004400, Phvul.002G006100, Phvul.002G006700, and Phvul.002G007100 have been reported to be involved in the morphology of seed and related tissues. Interestingly, the significantly associated SNP S1_53011276 was positioned within an exon of Phvul.002G007100. Although this does not necessarily indicate a cause–effect relationship, this gene is a good candidate since it encodes a SUPERMAN transcriptional regulator, one of the best characterized TFIIIA zinc finger proteins associated with plant development, including leaf, shoot, and floral organ morphogenesis and gametogenesis. According to the atlas, strong expression of this gene was not observed, and it was noticeably expressed only in late seed development (Supplementary Tables 1 and 2) and thus had high negative *Z*-scores compared to the whole gene set (Supplementary Table 2). Similarly, Huang *et al.* (2006) indicated that a SUPERMAN-like gene, namely *GmZFP1,* was intronless and expressed principally in the late developing seeds and reproductive organs of soybean. The authors further suggested that this gene might act through a MADS-box transcription factor-mediated signal network, as occurs in *Arabidopsis*. In the same LD block, Phvul.002G004400 was consistently expressed at the initial stage of seed development (SH) and belongs to a protein family recognized as a pentatricopeptide repeat-containing protein, a modular RNA-binding protein which intermediates numerous aspects of gene expression, such as RNA processing, splicing, editing, stability, and translation. Already associated with several phenotypes, this family is recognized as an important player in seed development in various crops (Gutiérrez-Marcos *et al.* 2007; Li *et al.* 2014; Verma *et al.* 2015; Lyu *et al.* 2020). In maize, empty pericarp5 and small kernel 1 mutants, both encoding pentatricopeptide repeat proteins, exhibited embryo abortion, small endosperm, and stunted seed development in the early stages (Liu *et al.* 2013b; Li *et al.* 2014; Qi *et al.* 2017). Furthermore, many other related genes have also been reported as candidates for seed size and weight regulation in genetic mapping studies of many crops (Zhou *et al.* 2017; Du *et al.* 2019), including Fabaceae (Verma *et al.* 2015). Phvul.002G006100, predicted as a protein phosphatase 2C 29-like gene, exhibited remarkable expressed in all 8 evaluated tissues. Phosphatase 2C proteins are crucial negative regulators of ABA signaling (Bai *et al.* 2013), and have been reported in soybean as important regulators of seed weight and size. Lu *et al.* (2017) used whole-genome sequencing of a recombinant inbred line population to map QTLs for seed weight, and noticed that a phosphatase 2C-1 allele from wild soybean had a marked effect in increasing seed weight/size, an observation also validated in transgenic plants. Finally, Phvul.002G006700, an AT-hook motif nuclear-localized protein 8, exhibited strong expression for FY, PH, and tissues at the 3 seed stages. For instance, Sharma Koirala and Neff (2020) showed that an AT-hook motif (AtSOB3-D) modulates seed size as well as hypocotyl length in *Camelina sativa* and *A*. *thaliana*.

Associated with LENGTH, PERIM, and CIRC, the polymorphism of S1_325844152 is located inside an exon of Phvul.008G020600, a crowded nuclei 1 protein, which exhibited the strongest absolute expression and highest *Z*-scores in most tissues considered herein, especially in the flowering and early developmental stages. Although this gene has not yet been reported as a direct modulator of seed size, the control it exercises over nuclear cell size was detailed (Wang *et al.* 2013), as well as its role in ABA-controlled seed germination (Zhao *et al.* 2016). In the same genomic haplotype block, Phvul.008G019500, a protein *Mei2*-like 4 isoform, is noteworthy for its strong expression in early-stage seed development (SH). *Mei2*-like proteins with conserved RNA recognition motifs have been identified in several plant species and seem to play a role in plant development (Anderson *et al.* 2004). In rice, an *Mei2*-like 4 protein (OML4) is phosphorylated by a glycogen synthase kinase 2 and negatively controls seed size and weight. Thus, loss of function of OML4 mutants produces larger and heavier seeds, while the overexpression of this gene leads to smaller, lighter grains (Lyu *et al.* 2020; Zhang 2020). As observed in the association of S1_53011276 with WIDTH and AREA, this region comprises genes encoding for pentatricopeptide repeat-containing proteins (Phvul.008G022600, Phvul.008G023900, Phvul.008G024300, and Phvul.008G024400), in which Phvul.008G022600 and Phvul.008G024400 were abundantly expressed in all the tissues investigated.

The region containing the association between AREA and S1_379992973, a putative B3 domain-containing protein (Phvul.008G244300), exhibited low transcriptional levels in the 8 tissues considered herein. The plant-specific B3 superfamily encompasses well-studied protein families, including prominent transcriptional elements, such as auxin response factors (Swaminathan *et al.* 2008). Transcription factors associated with this domain have been proved to be involved in activating and repressing seed development and grain filling in several crops and model species (Guo *et al.* 2013; Peng and Weselake 2013; Ahmad *et al.* 2019; Yang *et al.* 2021). Finally, an association between SNP S1_507205534 and LENGTH was found in an exonic region of a ribosomal protein L11 methyltransferase gene (Phvul.011G163100). In this region, a cup-shaped cotyledon 2-like gene (Phvul.011G160400) has a homolog previously reported to be involved in ovule development (Gonçalves *et al.* 2015). Aida *et al.* (1997) studied mutations in 2 homologs of this gene and found defects in the separation of cotyledons that can lead to notable modifications in seed shape.

Although GWAS has been very widely used to investigate the genetic control of several important common bean traits (see González *et al.* 2017; Assefa *et al.* 2019), including seed color (McClean *et al.* 2018; de Almeida *et al.* 2021), mineral content (Katuuramu *et al.* 2018; Gunjača *et al.* 2021), cooking time (Diaz *et al.* 2020), and flavor (Bassett *et al.* 2021), as far as we know ours is the first study to combine high-density genotyping and high-throughput image-based seed analysis to perform association mapping and dissect the genetic architecture of both seed size and shape in common bean. Our findings indicate that 4 genomic regions could explain a substantial amount of the phenotypic variation. From a practical standpoint, this study provides breeders with SNP markers that, given the concordance with the literature, are very good resources for use in marker-assisted selection. Moreover, several genes were pinpointed as interesting candidates underlying phenotypic variation in LENGTH, WIDTH, AREA, PERIM, and CIRC. These genes are promising choices for functional analysis, e.g. gene editing, which could validate their influence on seed shape and size in common bean and other related crops.

## Data availability

GBS data that support the findings of this study are available in the GenBank database, BioSample SAMN05513252 and SAMN05513251, included in BioProject PRJNA336556 (https://www.ncbi.nlm.nih.gov/bioproject/336556 [accessed 2020 Sep 10]). The genotypic information is available in Diniz *et al.* (2018) (https://www.mdpi.com/2073-4425/10/1/5#supplementary [accessed 2020 Sep 10], Supplementary Table 3). The phenotypic dataset generated and analyzed during the current study are included in Supplementary Files 1–3. The source codes employed in this study are included in Supplementary File 4.

Supplemental material is available at *G3* online.

## Supplementary Material

jkac048_File_S1

jkac048_File_S2

jkac048_File_S3

jkac048_File_S4

jkac048_Table_S1

jkac048_Table_S2

jkac048_Supplemental_Figures_Tables_and_Legends

## References

[jkac048-B1] Adams MW. Basis of yield component compensation in crop plants with special reference to the field bean, *Phaseolus vulgaris*. Crop Sci. 1967;7(5):505–510. doi:10.2135/cropsci1967.0011183X000700050030x.

[jkac048-B2] Ahmad B , Zhang S, Yao J, Rahman MU, Hanif M, Zhu Y, Wang X. Genomic organization of the B3-domain transcription factor family in grapevine (*Vitis vinifera* L.) and expression during seed development in seedless and seeded cultivars. Int J Mol Sci. 2019;20(18):4553.doi:10.3390/ijms20184553. PMC6770561 31540007

[jkac048-B3] Aida M , Ishida T, Fukaki H, Fujisawa H, Tasaka M. Genes involved in organ separation in *Arabidopsis*: an analysis of the cup-shaped cotyledon mutant. Plant Cell. 1997;9(6):841–857. doi:10.1105/tpc.9.6.841. 9212461 10.1105/tpc.9.6.841 PMC156962

[jkac048-B4] Akibode CS , Maredia MK. Global and Regional Trends in Production, Trade and Consumption of Food Legume Crops. East Lansing, MI, USA: Department of Agricultural, Food and Resource Economics, Michigan State University; 2011. doi:10.22004/ag.econ.136293.

[jkac048-B6] Anderson GH , Alvarez NDG, Gilman C, Jeffares DC, Trainor VCW, Hanson MR, Veit B. Diversification of genes encoding *Mei2*-like RNA binding proteins in plants. Plant Mol Biol. 2004;54(5):653–670. doi:10.1023/B:PLAN.0000040819.33383.b6. 15356386

[jkac048-B7] Assefa T , Mahama AA, Brown AV, Cannon EK, Rubyogo JC. A review of breeding objectives, genomic resources, and marker-assisted methods in common bean (*Phaseolus vulgaris* L.). Mol. Breed. 2019;39:1–23. doi:10.1007/s11032-018-0920-0.

[jkac048-B8] Bai G , Yang D-H, Zhao Y, Ha S, Yang F, Ma J, Gao X-S, Wang Z-M, Zhu J-K. Interactions between soybean ABA receptors and type 2C protein phosphatases. Plant Mol Biol. 2013;83(6):651–664. doi:10.1007/s11103-013-0114-4. 23934343 PMC3834219

[jkac048-B9] Bassett A , Kamfwa K, Ambachew D, Cichy K. Genetic variability and genome-wide association analysis of flavor and texture in cooked beans (*Phaseolus vulgaris* L.). Theor Appl Genet. 2021;134(3):959–978. doi:10.1007/s00122-020-03745-3. 33388888 PMC7925484

[jkac048-B10] Beaver JS , Osorno JM. Achievements and limitations of contemporary common bean breeding using conventional and molecular approaches. Euphytica. 2009;168(2):145–175. doi:10.1007/s10681-009–9911-x.

[jkac048-B11] Bellucci E , Bitocchi E, Rau D, Rodriguez M, Biagetti E, Giardini A, Attene G, Nanni L, Papa, R, et alGenomics of origin, domestication and evolution of *Phaseolus vulgaris*. In: R Tuberosa, A Graner, E Frison, editors. Genomics of Plant Genetic Resources. Dordrecht: Springer Netherlands; 2014. p. 483–507. doi:10.1007/978-94-007-7572-5_20.

[jkac048-B14] Bitocchi E , Rau D, Bellucci E, Rodriguez M, Murgia ML, Gioia T, Santo D, Nanni L, Attene G, Papa R, et al Beans (*Phaseolus* ssp.) as a model for understanding crop evolution. Front Plant Sci. 2017;8:722–730. doi:10.3389/fpls.2017.00722. 28533789 PMC5420584

[jkac048-B15] Blair MW , Cortés AJ, Farmer AD, Huang W, Ambachew D, Penmetsa RV, Carrasquilla-Garcia N, Assefa T, Cannon SB. Uneven recombination rate and linkage disequilibrium across a reference SNP map for common bean (*Phaseolus vulgaris* L.). PLoS One. 2018;13(3):e0189597.doi:10.1371/journal.pone.0189597. 29522524 PMC5844515

[jkac048-B16] Blair MW , Díaz LM, Buendía HF, Duque MC. Genetic diversity, seed size associations and population structure of a core collection of common beans (*Phaseolus vulgaris* L.). Theor Appl Genet. 2009;119(6):955–972. doi:10.1007/s00122-009-1064-8. 19688198

[jkac048-B17] Blair MW , Gonza LF, Kimani PM, Butare L. Genetic diversity, inter-gene pool introgression and nutritional quality of common beans (*Phaseolus vulgaris* L.) from Central Africa. Theor Appl Genet. 2010;121(2):237–248. doi:10.1007/s00122-010-1305-x. 20224891 PMC2886139

[jkac048-B18] Blair MW , Iriarte G, Beebe S. QTL analysis of yield traits in an advanced backcross population derived from a cultivated Andean × wild common bean (*Phaseolus vulgaris* L.) cross. Theor Appl Genet. 2006;112(6):1149–1163. doi:10.1007/s00122-006-0217-2. 16432734

[jkac048-B19] Broughton WJ , Hernández G, Blair M, Beebe S, Gepts P, Vanderleyden J. Beans (*Phaseolus* spp.) – model food legumes. Plant Soil. 2003;252(1):55–128. doi:10.1023/A:1024146710611.

[jkac048-B20] Butler DG , Cullis BR, Gilmour AR, Gogel BJ. Asreml–R Reference Manual. Brisbane (The State of Queensland): Department of Primary Industries and Fisheries; 2009.

[jkac048-B21] Chacón-Sánchez MI. The domestication syndrome in *Phaseolus* crop plants: a review of two key domestication traits. In: P Pontarotti, editor. Origin and Evolution of Biodiversity. 1st edn. Cham: Springer International Publishing; 2018. p. 37–59. doi:10.1007/978-3-319-95954-2_3.

[jkac048-B22] Cheadle C , Vawter MP, Freed WJ, Becker KG. Analysis of microarray data using Z score transformation. J. Mol. Diagnostics. 2003;5(2):73–81. doi:10.1016/S1525-1578(10)60455-2. PMC1907322 12707371

[jkac048-B23] Checa OE , Blair MW. Inheritance of yield-related traits in climbing beans (*Phaseolus vulgaris* L.). Crop Sci. 2012;52(5):1998–2013. doi:10.2135/cropsci2011.07.0368.

[jkac048-B24] Conesa A , Götz S, García-Gómez JM, Terol J, Talón M, Robles M. Blast2GO: a universal tool for annotation, visualization and analysis in functional genomics research. Bioinformatics. 2005;21(18):3674–3676. doi:10.1093/bioinformatics/bti610. 16081474

[jkac048-B25] de Almeida CP , Santos IL, de Carvalho Paulino JF, Barbosa CCF, Pereira CCA, Carvalho CRL, de Moraes Cunha Gonçalves G, Song Q, Carbonell SAM, Chiorato AF, et al Genome-wide association mapping reveals new loci associated with light-colored seed coat at harvest and slow darkening in carioca beans. BMC Plant Biol. 2021;21(1):343–357. doi:10.1186/s12870-021-03122-2. 34284717 PMC8290572

[jkac048-B26] Delfini J , Moda-Cirino V, dos Santos Neto J, Ruas PM, Sant’Ana GC, Gepts P, Gonçalves LSA. Population structure, genetic diversity and genomic selection signatures among a Brazilian common bean germplasm. Sci Rep. 2021;11(1):12.doi:10.1038/s41598-021–82437-4. 33536468 10.1038/s41598-021-82437-4 PMC7859210

[jkac048-B27] Diaz S , Ariza-Suarez D, Ramdeen R, Aparicio J, Arunachalam N, Hernandez C, Diaz H, Ruiz H, Piepho H-P, Raatz B. Genetic architecture and genomic prediction of cooking time in common bean (*Phaseolus vulgaris* L.). Front Plant Sci. 2020;11:622213.doi:10.3389/fpls.2020.622213. 33643335 PMC7905357

[jkac048-B28] Diniz AL , Giordani W, Costa ZP, Margarido GRA, Perseguini JMKC, Benchimol-Reis LL, Chiorato AF, Garcia AAF, Vieira MLC. Evidence for strong kinship influence on the extent of linkage disequilibrium in cultivated common beans. Genes. 2018;10(1):5.doi:10.3390/genes10010005. PMC6356217 30583474

[jkac048-B29] Du B , Wang Q, Sun G, Ren X, Cheng Y. Mapping dynamic QTL dissects the genetic architecture of grain size and grain filling rate at different grain-filling stages in barley. Sci. Rep. 2019;9:1–16. doi:10.1038/s41598-019-53620-5. 31827117 PMC6906516

[jkac048-B30] Elshire RJ , Glaubitz JC, Sun Q, Poland JA, Kawamoto K, Buckler ES, Mitchell SE. A robust, simple genotyping-by-sequencing (GBS) approach for high diversity species. PLoS One. 2011;6(5):e19379.doi:10.1371/journal.pone.0019379. 21573248 PMC3087801

[jkac048-B31] FAO and AfricaSeeds. Seeds Toolkit-Module 3: Seed Quality Assurance, 3nd edn. Rome: Food & Agriculture Org.; 2018.

[jkac048-B32] FAOSTAT. 2018. [accessed 2020 May 14]. http://www.fao.org/faostat/en/#data/QC.

[jkac048-B33] Fıratlıgil-Durmuş E , Šárka E, Bubník Z, Schejbal M, Kadlec P. Size properties of legume seeds of different varieties using image analysis. J. Food Eng. 2010;99(4):445–451. doi:10.1016/j.jfoodeng.2009.08.005.

[jkac048-B34] Flint-Garcia SA , Thornsberry JM, Buckler ES. Structure of linkage disequilibrium in plants. Annu Rev Plant Biol. 2003;54:357–374. doi:10.1146/annurev.arplant.54.031902.134907. 14502995

[jkac048-B35] Gavazzi G , Sangiorgio S. Seed size: an important yield component. In: R Pilu, G Gavazzi, editors. More Food: road to Survival. 1st edn. United Arab Emirates: Bentham Books; 2017. p. 143–167.

[jkac048-B36] Geng Q , Wang L, Wu J, Wang S. QTL Mapping for seed size and shape in common bean. Acta Agron. Sin. 2017;43(8):1149–1160. doi: 10.3724/SP.J.1006.2017.01149.

[jkac048-B37] Gepts P. Crop domestication as a long-term selection experiment. Plant Breed. Rev. 2004;24:1–44. doi: 10.1002/9780470650288.

[jkac048-B38] Gepts P , Aragão FJL, Barros E. D, Blair MW, Brondani R, et alGenomics of *Phaseolus* beans, a major source of dietary protein and micronutrients in the tropics. In: PH Moore, R Ming, editors. Genomics of Tropical Crop Plants. New York (NY): Springer; 2008. p. 113–143. doi:10.1007/978-0-387-71219-2_5.

[jkac048-B39] Gepts P , Bliss FA. Dissemination pathways of common bean (*Phaseolus vulgaris*, Fabaceae) deduced from phaseolin electrophoretic variability. II. Europe and Africa. Econ Bot. 1988;42(1):86–104. doi:10.1007/BF02859038.

[jkac048-B40] Gepts P , Osborn TC, Rashka K, Bliss FA. Phaseolin-protein variability in wild forms and landraces of the common bean (*Phaseolus vulgaris*): Evidence for multiple centers of domestication. Econ Bot. 1986;40(4):451–468. doi:10.1007/BF02859659.

[jkac048-B41] Geravandi M , Cheghamirza K, Farshadfar E, Gepts P. QTL analysis of seed size and yield-related traits in an inter-genepool population of common bean (*Phaseolus vulgaris* L.). Sci. Hortic. 2020;274(109678):109678.doi:10.1016/j.scienta.2020.109678Get.

[jkac048-B42] Giordani W , Gama HC, Chiorato AF, Marques JPR, Huo H, Benchimol‐Reis LL, Camargo LEA, Garcia AAF, Vieira MLC. Genetic mapping reveals complex architecture and candidate genes involved in common bean response to *Meloidogyne incognita* infection. Plant Genome. 2021;e20161.doi:10.1002/tpg2.20161. 34806826 PMC12807100

[jkac048-B43] Gonçalves B , Hasson A, Belcram K, Cortizo M, Morin H, Nikovics K, Vialette-Guiraud A, Takeda S, Aida M, Laufs P, et al A conserved role for CUP‐SHAPED COTYLEDON genes during ovule development. Plant J. 2015;83(4):732–742. doi:10.1111/tpj.12923. 26119568

[jkac048-B44] Gunjača J , Carović-Stanko K, Lazarević B, Vidak M, Petek M, Liber Z, Šatović Z. Genome-wide association studies of mineral content in common bean. Front Plant Sci. 2021;12:636484.doi:10.3389/fpls.2021.636484. 33763096 PMC7982862

[jkac048-B45] González AM , Yuste-Lisbona FJ, Fernández-Lozano A, Lozano R, Santalla M. Genetic mapping and QTL analysis in common bean. In: M Pérez de la Vega, M Santalla, F Marsolais, editors. The Common Bean Genome. Cham: Springer International Publishing; 2017. p. 69–107. doi:10.1007/978-3-319-63526-2_4.

[jkac048-B46] Guo X , Hou X, Fang J, Wei P, Xu B, Chen M, Feng Y, Chu C. The rice GERMINATION DEFECTIVE 1, encoding a B3 domain transcriptional repressor, regulates seed germination and seedling development by integrating GA and carbohydrate metabolism. Plant J. 2013;75(3):403–416. doi:10.1111/tpj.12209. 23581288 PMC3813988

[jkac048-B47] Gutiérrez-Marcos JF , Dal Prà M, Giulini A, Costa LM, Gavazzi G, Cordelier S, Sellam O, Tatout C, Paul W, Perez P, et al *empty pericarp4* encodes a mitochondrion-targeted pentatricopeptide repeat protein necessary for seed development and plant growth in maize. Plant Cell. 2007;19(1):196–210. doi:10.1105/tpc.105.039594. 17259266 PMC1820960

[jkac048-B48] Guzmán-Maldonado SH , Martínez O, Acosta-Gallegos JA, Guevara-Lara F, Paredes-López O. Putative quantitative trait loci for physical and chemical components of common bean. Crop Sci. 2003;43(3):1029–1035. doi:10.2135/cropsci2003.1029.

[jkac048-B49] Herron SA , Rubin MJ, Ciotir C, Crews TE, Van Tassel DL, Miller AJ. Comparative analysis of early life stage traits in annual and perennial *Phaseolus* crops and their wild relatives. Front Plant Sci. 2020;11:34–44. doi:10.3389/fpls.2020.00034. 32210978 PMC7076113

[jkac048-B50] Huang F , Chi Y, Meng Q, Gai J, Yu D. *GmZFP1* encoding a single zinc finger protein is expressed with enhancement in reproductive organs and late seed development in soybean (*Glycine max*). Mol Biol Rep. 2006;33(4):279–285. doi:10.1007/s11033-006-9012-z. 17077988

[jkac048-B51] Huang X , Han B. Natural variations and genome-wide association studies in crop plants. Annu Rev Plant Biol. 2014;65:531–551. doi:10.1146/annurev-arplant-050213-035715. 24274033

[jkac048-B52] Huang X , Wei X, Sang T, Zhao Q, Feng Q, Zhao Y, Li C, Zhu C, Lu T, Zhang Z, et al Genome-wide association studies of 14 agronomic traits in rice landraces. Nat Genet. 2010;42(11):961–967. doi:10.1038/ng.695. 20972439

[jkac048-B53] Johannsen W. The genotype conception of heredity. Am Nat. 1911;45(531):129–159. 10.1093/ije/dyu063 PMC4258772 24691957

[jkac048-B55] Kelly JD , Miklas PN. The role of RAPD markers in breeding for disease resistance in common bean. Mol Breed. 1998;4(1):1–11. doi:10.1023/A:1009612002144.

[jkac048-B57] Koinange EM , Singh SP, Gepts P. Genetic control of the domestication syndrome in common bean. Crop Sci. 1996;36(4):1037–1045. doi:10.2135/cropsci1996.0011183X003600040037x.

[jkac048-B58] Korte A , Farlow A. The advantages and limitations of trait analysis with GWAS: a review. Plant Methods. 2013;9:29. doi:10.1186/1746–4811-9–29. 23876160 10.1186/1746-4811-9-29 PMC3750305

[jkac048-B59] Kumar P , Chatli MK, Mehta N, Singh P, Malav OP, Verma AK. Meat analogues: health promising sustainable meat substitutes. Crit Rev Food Sci Nutr. 2017;57(5):923–932. doi:10.1080/10408398.2014.939739. 25898027 10.1080/10408398.2014.939739

[jkac048-B60] Katuuramu DN , Hart JP, Porch TG, Grusak MA, Glahn RP, Cichy KA. Genome-wide association analysis of nutritional composition-related traits and iron bioavailability in cooked dry beans (*Phaseolus vulgaris* L.). Mol Breeding. 2018;38(4):44–62. doi:10.1007/s11032-018-0798-x.

[jkac048-B61] Kwak M , Gepts P. Structure of genetic diversity in the two major gene pools of common bean (*Phaseolus vulgaris* L., Fabaceae). Theor Appl Genet. 2009;118(5):979–992. doi:10.1007/s00122-008-0955-4. 19130029

[jkac048-B62] Lander ES , Botstein D. Mapping Mendelian factors underlying quantitative traits using RFLP linkage maps. Genetics. 1989;121(1):185–199. doi:10.1093/genetics/121.1.185. 2563713 PMC1203601

[jkac048-B63] Lei L , Wang L, Wang S, Wu J. Marker-trait association analysis of seed traits in accessions of common bean (*Phaseolus vulg*aris L.) in China. Front Genet. 2020;11:698–710. doi:10.3389/fgene.2020.00698. 32714377 10.3389/fgene.2020.00698 PMC7344293

[jkac048-B64] Li X-J , Zhang Y-F, Hou M, Sun F, Shen Y, Xiu Z-H, Wang X, Chen Z-L, Sun SSM, Small I, et al *small kernel 1* encodes a pentatricopeptide repeat protein required for mitochondrial nad7 transcript editing and seed development in maize (*Zea mays*) and rice (*Oryza sativa*). Plant J. 2014;79(5):797–809. doi:10.1111/tpj.12584. 24923534

[jkac048-B65] Lipka AE , Tian F, Wang Q, Peiffer J, Li M, Bradbury PJ, Gore MA, Buckler ES, Zhang Z. GAPIT: genome association and prediction integrated tool. Bioinformatics. 2012;28(18):2397–2399. doi:10.1093/bioinformatics/bts444. 22796960

[jkac048-B66] Liu S , Wang X, Wang H, Xin H, Yang X, Yan J, Li J, Tran L-SP, Shinozaki K, Yamaguchi-Shinozaki K, et al Genome-wide analysis of *ZmDREB* genes and their association with natural variation in drought tolerance at seedling stage of *Zea mays* L. PLoS Genet. 2013a;9(9):e1003790. doi:10.1371/journal.pgen.1003790. 24086146 PMC3784558

[jkac048-B67] Liu Y-J , Xiu Z-H, Meeley R, Tan B-C. *empty pericarp5* encodes a pentatricopeptide repeat protein that is required for mitochondrial RNA editing and seed development in maize. Plant Cell. 2013b;25(3):868–883. doi:10.1105/tpc.112.106781. 23463776 PMC3634694

[jkac048-B68] Logozzo G , Donnoli R, Macaluso L, Papa R, Knüpffer H, Zeuli PS. Analysis of the contribution of Mesoamerican and Andean gene pools to European common bean (*Phaseolus vulgaris* L.) germplasm and strategies to establish a core collection. Genet Resour Crop Evol. 2007;54(8):1763–1779. doi:10.1007/s10722-006-9185-2.

[jkac048-B69] Lu X , Xiong Q, Cheng T, Li Q-T, Liu X-L, Bi Y-D, Li W, Zhang W-K, Ma B, Lai Y-C, et al A PP2C-1 allele underlying a quantitative trait locus enhances soybean 100-seed weight. Mol Plant. 2017;10(5):670–684. doi:10.1016/j.molp.2017.03.006. 28363587

[jkac048-B70] Lyu J , Wang D, Duan P, Liu Y, Huang K, Zeng D, Zhang L, Dong G, Li Y, Xu R, et al Control of grain size and weight by the GSK2-LARGE1/OML4 pathway in rice. Plant Cell. 2020;32(6):1905–1918. doi:10.1105/tpc.19.00468. 32303659 PMC7268794

[jkac048-B71] Mamidi S , Rossi M, Moghaddam SM, Annam D, Lee R, Papa R, McClean PE. Demographic factors shaped diversity in the two gene pools of wild common bean *Phaseolus vulgaris* L. Heredity. 2013;110(3):267–276. doi:10.1038/hdy.2012.82. 23169559 PMC3668653

[jkac048-B72] Mazhar KARA , Sayinci B, Elkoca E, et al Seed size and shape analysis of registered common bean (*Phaseolus vulgaris* L.) cultivars in Turkey using digital photography. J Agric Sci. 2013;19:219–234.

[jkac048-B73] McClean PE , Bett KE, Stonehouse R, Lee R, Pflieger S, Moghaddam SM, Geffroy V, Miklas P, Mamidi S. White seed color in common bean (*Phaseolus vulgaris*) results from convergent evolution in the P (pigment) gene. New Phytol. 2018;219(3):1112–1123. doi:10.1111/nph.15259. 29897103

[jkac048-B74] Miklas PN , Kelly JD, Beebe SE, Blair MW. Common bean breeding for resistance against biotic and abiotic stresses: from classical to MAS breeding. Euphytica. 2006;147(1–2):105–131. doi:10.1007/s10681-006-4600-5.

[jkac048-B1001] Mir RR, Reynolds M, Pinto F, Khan MA, Bhat MA. High-throughput phenotyping for crop improvement in the genomics era. Plant Sci. 2019;282:60–72. doi:10.1016/j.plantsci.2019.01.007. 10.1016/j.plantsci.2019.01.007 31003612

[jkac048-B75] Mitchell-Olds T. Complex-trait analysis in plants. Genome Biol. 2010;11(4):113–115. doi:10.1186/gb-2010-11-4-113. 20409352 PMC2884532

[jkac048-B76] Motto M , Soressi GP, Salamini F. Seed size inheritance in a cross between wild and cultivated common beans (*Phaseolus vulgaris* L.). Genetica. 1978;49(1):31–36.

[jkac048-B77] Murube E , Campa A, Song Q, McClean P, Ferrreira JJ. Toward validation of QTLs associated with pod and seed size in common bean using two nested recombinant inbred line populations. Mol. Breed. 2020;40:1–17. doi:10.1007/s11032-019-1085-1.

[jkac048-B78] Nienhuis J , Singh SP. Combining ability analyses and relationships among yield, yield components, and architectural traits in dry bean. Crop Sci. 1986;26(1):21–27. doi:10.2135/cropsci1986.0011183X002600010005x

[jkac048-B79] O’Leary NA , Wright MW, Brister JR, Ciufo S, Haddad D, McVeigh R, Rajput B, Robbertse B, Smith-White B, Ako-Adjei D, et al Reference sequence (RefSeq) database at NCBI: current status, taxonomic expansion, and functional annotation. Nucleic Acids Res. 2016;44(D1):D733–745. doi:10.1093/nar/gkv1189. 26553804 PMC4702849

[jkac048-B80] OpenCV Developers Team. OpenCV reference manual. 2012. [accessed 2020 Sept 10]. https://docs.opencv.org/4.x/d4/db1/tutorial_documentation.html.

[jkac048-B81] O’Rourke JA , Iniguez LP, Fu F, Bucciarelli B, Miller SS, Jackson SA, McClean PE, Li J, Dai X, Zhao PX, et al An RNA-Seq based gene expression atlas of the common bean. BMC Genomics. 2014;15:866–817. doi:10.1186/1471-2164-15-866. 25283805 PMC4195886

[jkac048-B82] Park SO , Coyne DP, Jung G, Skroch PW, Arnaud-Santana E, Steadman JR, Ariyarathne HM, Nienhuis J. Mapping of QTL for seed size and shape traits in common bean. J Am Soc Hort Sci. 2000;125(4):466–475. doi:10.21273/JASHS.125.4.466.

[jkac048-B83] Peng FY , Weselake RJ. Genome-wide identification and analysis of the B3 superfamily of transcription factors in Brassicaceae and major crop plants. Theor Appl Genet. 2013;126(5):1305–1319. doi:10.1007/s00122-013-2054-4. 23377560

[jkac048-B84] Pérez-Vega E , Pañeda A, Rodríguez-Suárez C, Campa A, Giraldez R, Ferreira JJ. Mapping of QTLs for morpho-agronomic and seed quality traits in a RIL population of common bean (*Phaseolus vulgaris* L.). Theor Appl Genet. 2010;120(7):1367–1380. doi:10.1007/s00122-010-1261-5. 20084493

[jkac048-B85] Perseguini JMKC , Silva GMB, Rosa JRBF, Gazaffi R, Marçal JF, Carbonell SAM, Chiorato AF, Zucchi MI, Garcia AAF, Benchimol-Reis LL, et al Developing a common bean core collection suitable for association mapping studies. Genet Mol Biol. 2015;38(1):67–78. doi:10.1590/S1415-475738120140126. 25983627 PMC4415564

[jkac048-B86] Petrusán JI , Rawel H, Huschek G. Protein-rich vegetal sources and trends in human nutrition: a review. Curr Top Pept Protein Res. 2016;17:1–19.

[jkac048-B87] Petry N , Boy E, Wirth JP, Hurrell RF. Review: the potential of the common bean (*Phaseolus vulgaris*) as a vehicle for iron biofortification. Nutrients. 2015;7(2):1144–1173. doi:10.3390/nu7021144. 25679229 PMC4344581

[jkac048-B88] Qi W , Yang Y, Feng X, Zhang M, Song R. Mitochondrial function and maize kernel development requires Dek2, a pentatricopeptide repeat protein involved in nad1 mRNA splicing. Genetics. 2017;205(1):239–249. doi:10.1534/genetics.116.196105. 27815362 PMC5223505

[jkac048-B89] Rana JC , Sharma TR, Tyagi RK, Chahota RK, Gautam NK, Singh M, Sharma PN, Ojha SN. Characterisation of 4274 accessions of common bean (*Phaseolus vulgaris* L.) germplasm conserved in the Indian gene bank for phenological, morphological and agricultural traits. Euphytica. 2015;205(2):441–457. doi:10.1007/s10681-015–1406-3.

[jkac048-B90] Roll-Hansen N. The crucial experiment of Wilhelm Johannsen. Biol Philos. 1989;4(3):303–329. doi:10.1007/BF02426630.

[jkac048-B91] Sakai H , Krizek BA, Jacobsen SE, Meyerowitz EM. Regulation of SUP expression identifies multiple regulators involved in *Arabidopsis* floral meristem development. Plant Cell. 2000;12(9):1607–1618. doi:10.1105/tpc.12.9.1607. 11006335 PMC149073

[jkac048-B92] Sakai H , Medrano LJ, Meyerowitz EM. Role of SUPERMAN in maintaining *Arabidopsis* floral whorl boundaries. Nature. 1995;378(6553):199–203. doi:10.1038/378199a0. 7477325

[jkac048-B93] Santalla M , Monteagudo AB, González AM, De Ron AM. Agronomical and quality traits of runner bean germplasm and implications for breeding. Euphytica. 2004;135(2):205–215. doi:10.1023/B:EUPH.0000014912.07993.e7.

[jkac048-B94] Sax K , The association of size differences with seed-coat pattern and pigmentation in *Phaseolus vulgaris*. Genetics. 1923;8(6):552–560. 17246026 10.1093/genetics/8.6.552 PMC1200765

[jkac048-B95] Schmutz J , McClean PE, Mamidi S, Wu GA, Cannon SB, Grimwood J, Jenkins J, Shu S, Song Q, Chavarro C, et al A reference genome for common bean and genome-wide analysis of dual domestications. Nat Genet. 2014;46(7):707–713. doi:10.1038/ng.3008. 24908249 PMC7048698

[jkac048-B96] Segura V , Vilhjálmsson BJ, Platt A, Korte A, Seren Ü, Long Q, Nordborg M. An efficient multi-locus mixed-model approach for genome-wide association studies in structured populations. Nat Genet. 2012;44(7):825–830. doi:10.1038/ng.2314. 22706313 PMC3386481

[jkac048-B97] Sharma Koirala P , Neff MM. Improving seed size, seed weight and seedling emergence in *Camelina sativa* by overexpressing the Atsob3-6 gene variant. Transgenic Res. 2020;29(4):409–418. doi:10.1007/s11248-020-00208-9. 32748170

[jkac048-B98] Shaw N , Barak RS, Campbell RE, Kirmer A, Pedrini S, et al Seed use in the field: delivering seeds for restoration success. Restor. Ecol. 2020;28:276–285. doi:10.1111/rec.13210.

[jkac048-B99] Shin JH , Blay S, McNeney B, Graham J. LDheatmap: an R function for graphical display of pairwise linkage disequilibria between single nucleotide polymorphisms. J. Stat. Softw. 2006;16:1–10. doi:10.18637/jss.v016.c03.

[jkac048-B100] Singh DK , Singh DP, Singh SS. Studies of genetic variability, heritability and genetic advance for yield and related traits in French bean (*Phaseolus vulgaris* L.). J Pharmac. Phytoch. 2018;7:236–240.

[jkac048-B101] Singh SP , Gepts P, Debouck DG. Races of common bean (*Phaseolus vulgaris*, Fabaceae). Econ Bot. 1991a;45(3):379–396. doi:10.1007/BF02887079.

[jkac048-B102] Singh SP , Gutierrez JA, Molina A, Urrea C, Gepts P. Genetic diversity in cultivated common bean: II. Marker-based analysis of morphological and agronomic traits. Crop Sci. 1991b;31(1):23–29. doi:10.2135/cropsci1991.0011183X003100010005x.

[jkac048-B103] Singh SP , Schwartz HF. Breeding common bean for resistance to diseases: a review. Crop Sci. 2010;50(6):2199–2223. doi:10.2135/cropsci2009.03.0163.

[jkac048-B104] Swaminathan K , Peterson K, Jack T. The plant B3 superfamily. Trends Plant Sci. 2008;13(12):647–655. doi:10.1016/j.tplants.2008.09.006. 18986826 10.1016/j.tplants.2008.09.006

[jkac048-B105] Tanabata T , Shibaya T, Hori K, Ebana K, Yano M. SmartGrain: high-throughput phenotyping software for measuring seed shape through image analysis. Plant Physiol. 2012;160(4):1871–1880. doi:10.1104/pp.112.205120. 23054566 PMC3510117

[jkac048-B106] Varshney RK , Nayak SN, May GD, Jackson SA. Next-generation sequencing technologies and their implications for crop genetics and breeding. Trends Biotechnol. 2009;27(9):522–530. doi:10.1016/j.tibtech.2009.05.006. 19679362

[jkac048-B107] Verma S , Gupta S, Bandhiwal N, Kumar T, et al High-density linkage map construction and mapping of seed trait QTLs in chickpea (*Cicer arietinum* L.) using genotyping-by-sequencing (GBS). Sci Rep. 2015;5:1–14. doi:10.1038/srep17512. PMC4668357 26631981

[jkac048-B108] Wang H , Dittmer TA, Richards EJ. *Arabidopsis* CROWDED NUCLEI (CRWN) proteins are required for nuclear size control and heterochromatin organization. BMC Plant Biol. 2013;13(1):1–13. doi:10.1186/1471-2229-13-200. 24308514 PMC3922879

[jkac048-B109] White JW , González A. Characterization of the negative association between seed yield and seed size among genotypes of common bean. Field Crops Res. 1990;23(3–4):159–175. doi:10.1016/0378-4290(90)90052-D.

[jkac048-B110] Wright EM , Kelly JD. Mapping QTL for seed yield and canning quality following processing of black bean (*Phaseolus vulgaris* L.). Euphytica. 2011;179(3):471–484. doi:10.1007/s10681-011-0369-2.

[jkac048-B111] Wu J , Wang L, Fu J, Chen J, Wei S, Zhang S, Zhang J, Tang Y, Chen M, Zhu J, et al Resequencing of 683 common bean genotypes identifies yield component trait associations across a north–south cline. Nat Genet. 2020;52(1):118–125. doi:10.1038/s41588-019-0546-0. 31873299 10.1038/s41588-019-0546-0

[jkac048-B112] Xu Y , Li P, Yang Z, Xu C. Genetic mapping of quantitative trait loci in crops. Crop J. 2017;5(2):175–184. doi:10.1016/j.cj.2016.06.003.

[jkac048-B113] Yang T , Guo L, Ji C, Wang H, Wang J, Zheng X, Xiao Q, Wu Y. The B3 domain-containing transcription factor ZmABI19 coordinates expression of key factors required for maize seed development and grain filling. Plant Cell. 2021;33(1):104–128. doi:10.1093/plcell/koaa008. 33751093 PMC8136913

[jkac048-B114] Yuste-Lisbona FJ , González AM, Capel C, García-Alcázar M, Capel J, De Ron AM, Lozano R, Santalla M. Genetic analysis of single-locus and epistatic QTLs for seed traits in an adapted × nuña RIL population of common bean (*Phaseolus vulgaris* L.). Theor Appl Genet. 2014;127(4):897–912. doi:10.1007/s00122-014-2265-3. 24441949

[jkac048-B115] Zhang T. When less is more: GSK2-OML4 module negatively regulates grain size in rice. Plant Cell. 2020;32(6):1781.doi:10.1105/tpc.20.00219. 32303660 PMC7268792

[jkac048-B116] Zhang Z , Ersoz E, Lai C-Q, Todhunter RJ, Tiwari HK, Gore MA, Bradbury PJ, Yu J, Arnett DK, Ordovas JM, et al Mixed linear model approach adapted for genome-wide association studies. Nat Genet. 2010;42(4):355–360. doi:10.1038/ng.546. 20208535 PMC2931336

[jkac048-B117] Zhao K , Aranzana MJ, Kim S, Lister C, Shindo C, Tang C, Toomajian C, Zheng H, Dean C, Marjoram P, et al An *Arabidopsis* example of association mapping in structured samples. PLoS Genet. 2007;3(1):e4. doi:10.1371/journal.pgen.0030004. 17238287 PMC1779303

[jkac048-B118] Zhao W , Guan C, Feng J, Liang Y, Zhan N, Zuo J, Ren B. The Arabidopsis CROWDED NUCLEI genes regulate seed germination by modulating degradation of ABI5 protein. J Integr Plant Biol. 2016;58(7):669–678. doi:10.1111/jipb.12448. 26564029

[jkac048-B119] Zhou Q , Dong Y, Shi Q, Zhang L, Chen H, Hu C, Li Y. Verification and fine mapping of qGW1.05, a major QTL for grain weight in maize (*Zea mays* L.). Mol Genet Genomics. 2017;292(4):871–881. doi:10.1007/s00438-017-1318-0. 28405778

[jkac048-B120] Zhou Z , Jiang Y, Wang Z, Gou Z, Lyu J, Li W, Yu Y, Shu L, Zhao Y, Ma Y, et al Resequencing 302 wild and cultivated accessions identifies genes related to domestication and improvement in soybean. Nat Biotechnol. 2015;33(4):408–414. doi:10.1038/nbt.3096. 25643055

[jkac048-B121] Zhu C , Gore M, Buckler ES, Yu J. Status and prospects of association mapping in plants. Plant Genome. 2008;1(1):5–20. doi:10.3835/plantgenome2008.02.0089.

