# Supplementary material for: Genome-wide association studies dissect the genetic architecture of seed shape and size in common bean
Source: G3 (Bethesda). 2022 Feb 26;12(4):jkac048. doi: 10.1093/g3journal/jkac048 (PMC8982408; doi:10.1093/g3journal/jkac048)
Supplement: jkac048_Supplemental_Figures_Tables_and_Legends [file jkac048_supplemental_figures_tables_and_legends.docx]

**Supplementary Material**

**Manuscript Title**

Genome-wide association studies dissect the genetic architecture of seed shape and size in common bean

**Authorship**

Willian Giordani, Henrique Castro Gama, Alisson Fernando Chiorato, Antonio Augusto Franco Garcia, Maria Lucia Carneiro Vieira


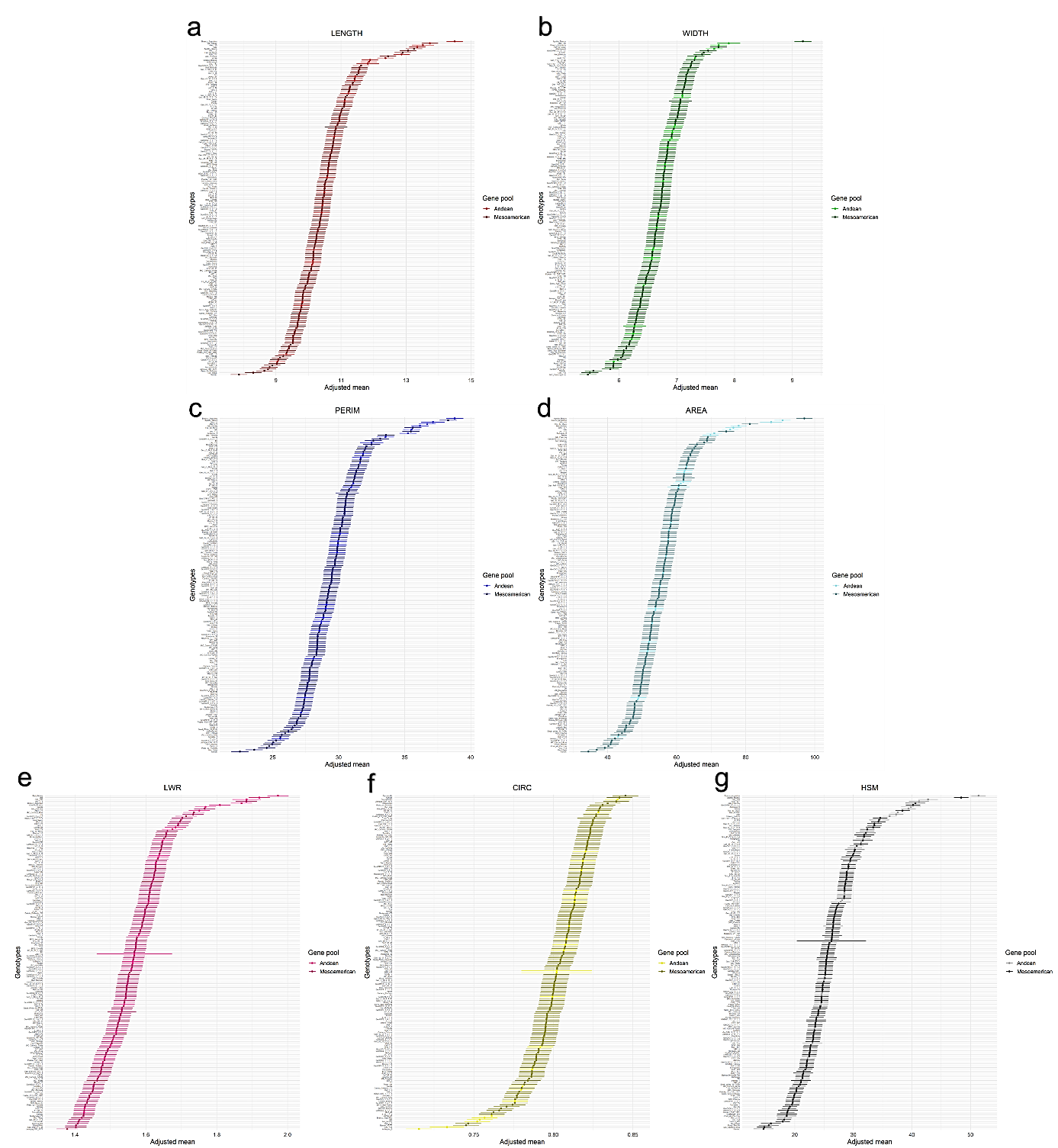


**Supplemental Figure S1**

Caterpillar plot of adjusted means with 95% confidence intervals for seven seed traits: seed length (a; LENGTH); width (b; WIDTH); perimeter (c; PERIM); projected area (d; AREA); length-to-width ratio (e; LWR); circularity (f; CIRC) and hundred seed mass (g; HSM), in 180 genotypes of the Andean (lighter dashes) and Mesoamerican (darker dashes) gene pools.


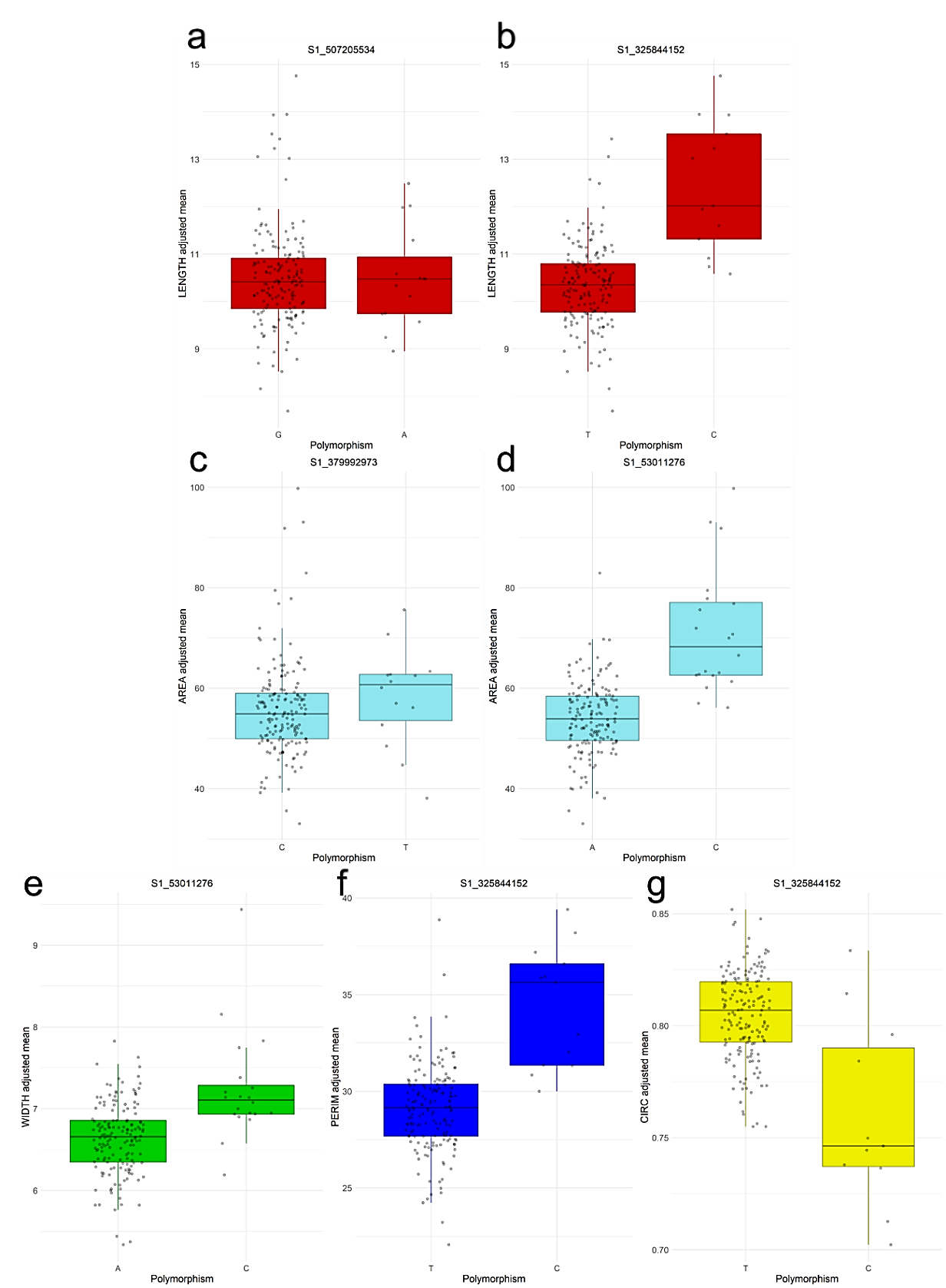


**Supplemental Figure S2**

Boxplots of the adjusted means of common bean accessions for SNPs significantly associated based on GWAS. Seed length (a and b; LENGTH) is represented in red; seed projected area (c and d; AREA) in light blue; seed width (e; WIDTH) in green; seed perimeter (f; PERIM) in blue; and circularity (g; CIRC) in yellow. No associations were detected for the remaining seed traits.


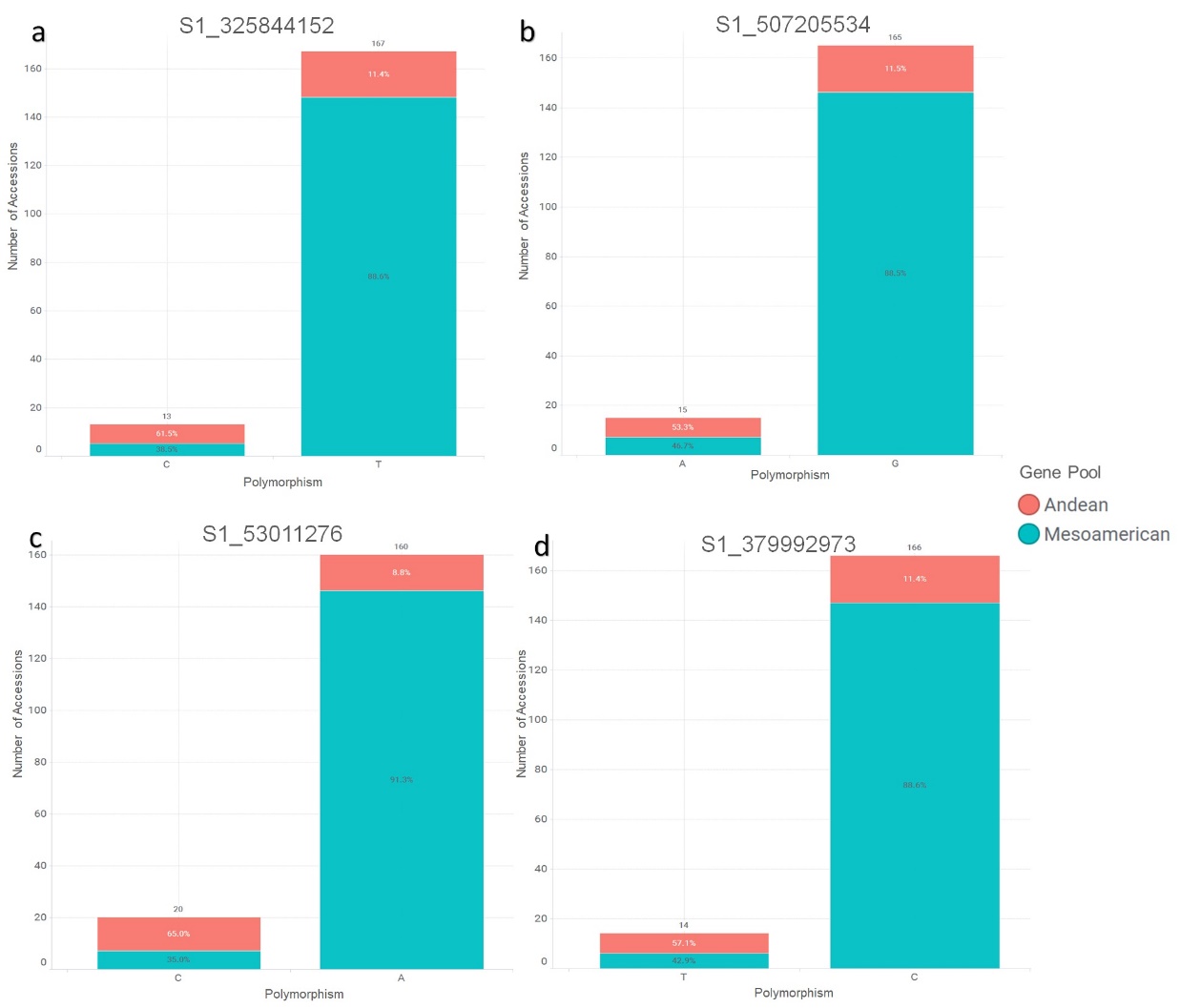


**Supplemental Figure S3**

Number of accessions and gene pool frequency for each polymorphism in significantly associated SNPs: S1_325844152 (a), S1_507205534 (b), S1_53011276 (c) and S1_379992973 (d).


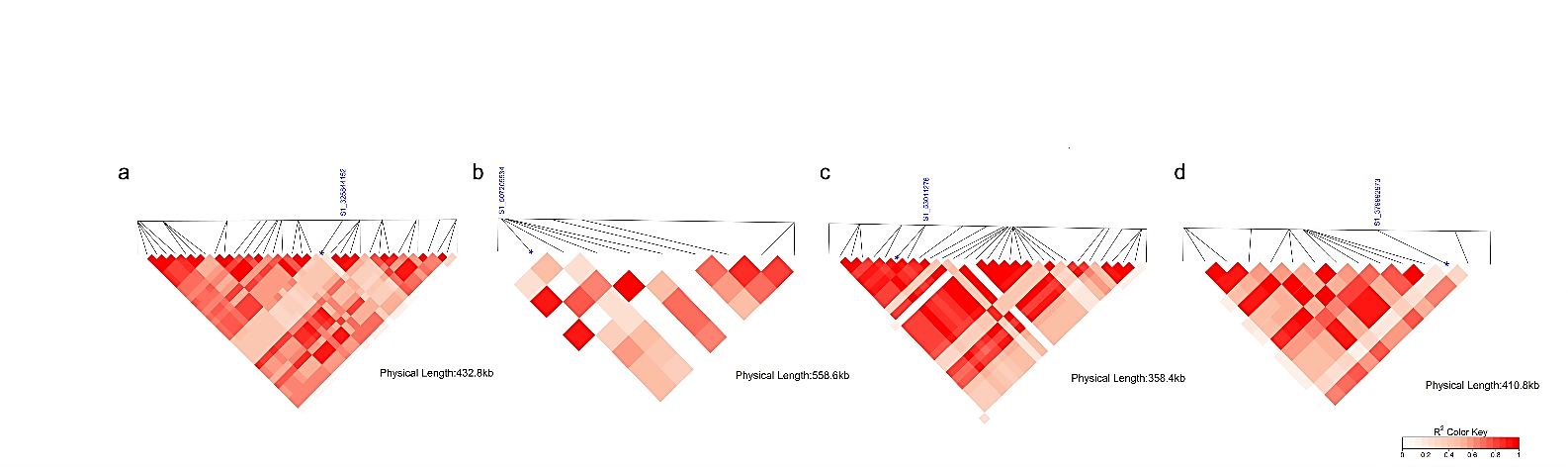


**Supplemental Figure S4**

Linkage disequilibrium (LD) analysis to define genomic regions for candidate gene discovery among significant SNPs: S1_325844152 (a), S1_507205534 (b), S1_53011276 (c) and S1_379992973 (d), and flanking markers in linkage disequilibrium with common bean seed traits. The heatmap indicates the degree of estimated LD.


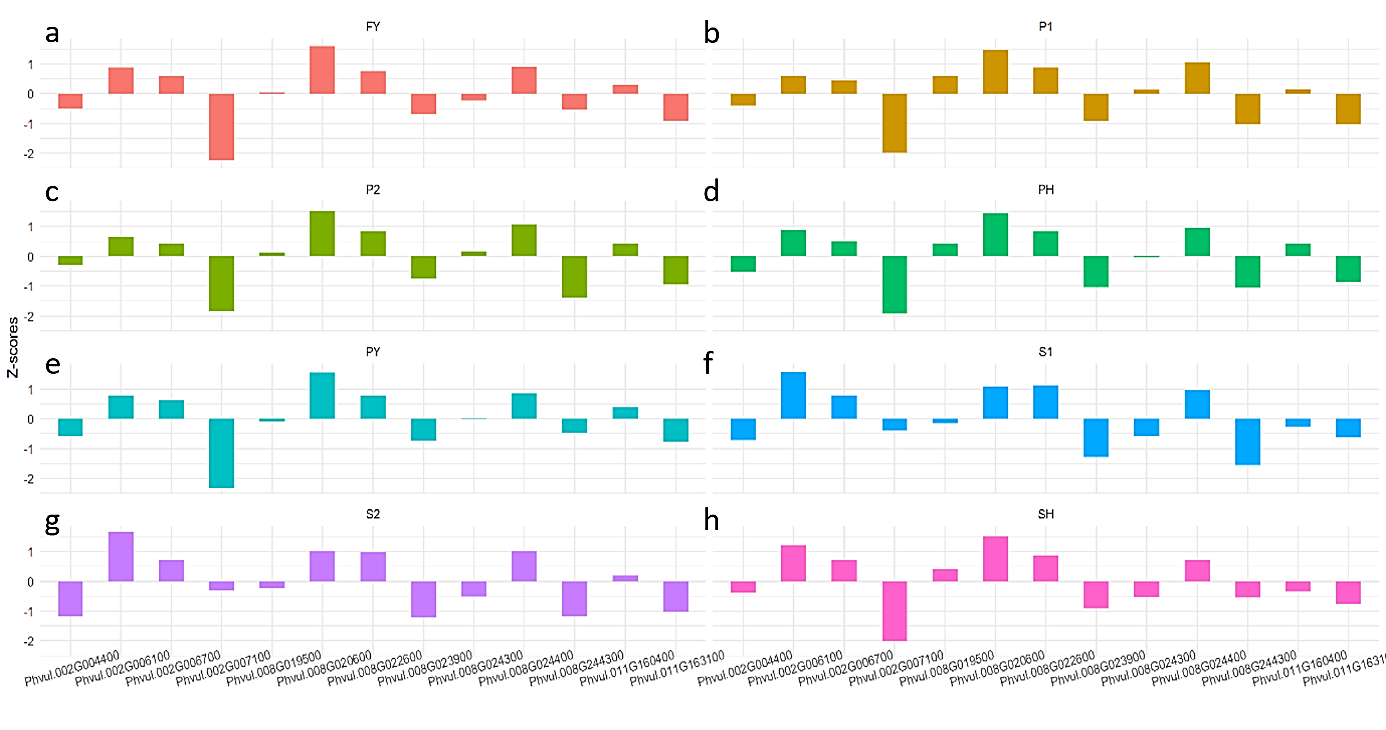


**Supplemental Figure S5**

Z-scores calculated from the absolute expressions of 13 common bean candidate genes for regulating common bean seed shape and size in eight tissues related to seed development according to the Common Bean Expression Atlas (O’Rourke et al. 2014). Young flowers (a; FY), pods between 10 and 11 cm long (b; P1), pods between 12 and 13 cm long (c; P2), pods approximately 9 cm long (d; PH), young pods (e; PY), stage 1 seeds (f; S1), stage 2 seeds (g; S2) and heart stage seeds (h; SH).

*Supplemental_Table_S1.xlsx*

**Supplemental Table S1**

Functional annotation and ontology terms predicted for candidate genes located in genomic regions significantly associated with common bean seed morphology.

*Supplemental_Table_S2.xlsx*

**Supplemental Table S2**

RPKM values obtained from the Common Bean Expression Atlas (O’Rourke et al. 2014) for 13 common bean candidate genes regulating seed shape and size in eight tissues related to seed development: young flowers (FY), young pods (PY), pods approximately 9 cm long (PH), pods between 10 and 11 cm long (P1), pods between 12 and 13 cm long (P2), heart stage seeds (SH), stage 1 seeds (S1), and stage 2 seeds (S2).

*Supplemental_File_S1.xlsx*

**Supplemental File S1**

Raw dataset from image-based seed phenotyping.

*Supplemental_File_S2.txt*

**Supplemental File S2**

Phenotypic BLUEs for the entire set of seed traits.

*Supplemental_File_S3.txt*

**Supplemental File S3**

Phenotypic BLUPs for the entire set of seed traits.

*Supplemental_File_S4.r*

**Supplemental File S4**

Source codes employed in the current study.
